# Characteristics of sensory neuronal groups in CGRP-cre-ER reporter mice: Comparison to Nav1.8-cre, TRPV1-cre and TRPV1-GFP mouse lines

**DOI:** 10.1371/journal.pone.0198601

**Published:** 2018-06-04

**Authors:** Mayur J. Patil, Anahit H. Hovhannisyan, Armen N. Akopian

**Affiliations:** 1 Departments of Endodontics, University of Texas Health Science Center at San Antonio, San Antonio, Texas, United States of America; 2 Departments of Pharmacology, University of Texas Health Science Center at San Antonio, San Antonio, Texas, United States of America; University of Southern California, UNITED STATES

## Abstract

Peptidergic sensory neurons play a critical role in nociceptive pathways. To precisely define the function and plasticity of sensory neurons in detail, new tools such as transgenic mouse models are needed. We employed electrophysiology and immunohistochemistry to characterize in detail dorsal root ganglion (DRG) neurons expressing an inducible CGRP^cre-ER^ (CGRP-cre^+^); and compared them to DRG neurons expressing Nav1.8^cre^ (Nav1.8-cre^+^), TRPV1^cre^ (TRPV1-cre^+^) and TRPV1-GFP (V1-GFP^+^). Tamoxifen effectively induced CGRP^cre-ER^ production in DRG. ≈87% of CGRP^cre-ER^-expressing neurons were co-labeled CGRP antibody. Three small and two medium-large-sized (5HT3a^+^/NPY2R^-^ and NPY2R^+^) neuronal groups with unique electrophysiological profiles were CGRP-cre^+^. Nav1.8-cre^+^ neurons were detected in all CGRP-cre^+^ groups, as well as in 5 additional neuronal groups: MrgprD^+^/TRPA1^-^, MrgprD^+^/TRPA1^+^, TRPV1^+^/CGRP^-^, vGLUT3^+^ and ≈30% of trkC^+^ neurons. Differences between TRPV1^cre^ and Nav1.8^cre^ reporters were that unlike TRPV1-cre^+^, Nav1.8-cre^+^ expression was detected in non-nociceptive vGLUT3^+^ and trkC^+^ populations. Many TRPV1-cre^+^ neurons did not respond to capsaicin. In contrast, V1-GFP^+^ neurons were in 4 groups, each of which was capsaicin-sensitive. Finally, none of the analyzed reporter lines showed *cre*-recombination in trkB^+^, calbindin^+^, 70% of trkC^+^ or parvalbumin^+^ neurons, which together encompassed ≈20% of Nav1.8-cre^-^ DRG neurons. The data presented here increases our knowledge of peptidergic sensory neuron characteristics, while showing the efficiency and specificity manipulation of peptidergic neurons by the CGRP^cre-ER^ reporter. We also demonstrate that manipulation of all C- and A-nociceptors is better achieved with TRPV1-cre reporter. Finally, the described approach for detailed characterization of sensory neuronal groups can be applied to a variety of reporter mice.

## Introduction

It is now recognized that somatosensory neurons are neurochemically, functionally and physiologically diverse [1,2,3]. This diversity allows for the detection of a wide range of sensory stimuli such as light touch, pressure, vibration, heat, cold and itch. With regards to pathological pain, it has been suggested that the roles of sensory neuronal groups are distinct [4]. Recent studies have utilized multiple markers such as MrgprA3, MrgprD, NPY2R, vGLUT3, trkB, trkC, calbindin (Calb) and parvalbumin (PV) for electrophysiological characterization of subsets of sensory neurons, and define their physiological functions [5,6,7,8,9,10,11]. Moreover, development of the next generation high-throughput and single-cell sequencing approaches has built transcription profiles for many sensory neuronal subsets/groups [1,7,12].

Sensory neurons involved in nociception and itch transmission are often divided into “peptidergic” and “non-peptidergic”. Peptidergic neurons are defined as calcitonin gene-related peptide-positive (CGRP^+^) [4]; and the majority express trkA [13]. Peptidergic neurons are important in triggering neurogenic inflammation [14,15,16]. The functional differences between peptidergic and non-peptidergic nociceptors and especially the myriad sub-groups of peptidergic nociceptors are not well known. In this respect, an effective transgenic mouse model and detailed characterization of peptidergic neuronal sub-groups could help us better understand the functions of peptidergic neurons in pain conditions and how they interact with immune and endocrine systems.

A mouse line with inducible *cre*-recombinase driven by the CGRP-α (CALCA) promotor (CGRP^cre/+-ER^) has been generated [17]. The aims of the present study were to generate comprehensive electrophysiological profile and anatomical characterization of neuronal groups/clusters visualized in DRG of CGRP^cre-ER^/TdTomato (labeled as CGRP-cre^+^) mice and compare them with properties of neuronal groups detected in DRG of Nav1.8^cre^/TdTomato (Nav1.8-cre^+^) [18], TRPV1^cre^/TdTomato (TRPV1-cre^+^) [19] and TRPV1-GFP (V1-GFP^+^) mice. Results of this study will advance our knowledge of peptidergic sensory neuron characteristic and offer an effective transgenic model for manipulation of peptidergic nociceptors. In addition, the described approach can be utilized in characterizing sensory neuronal groups expressing a variety of reporter mice; and sensory neuronal groups innervated by a range of tissues.

## Material and methods

### Mouse reporter lines

All animal experiments conformed to APS's Guiding Principles in the Care and Use of Vertebrate Animals in Research and Training. We also followed guidelines issued by the National Institutes of Health (NIH) and the Society for Neuroscience (SfN) to minimize the numbers of animals used and their suffering. Protocols specifically used in these studies (20150100AR and 20150109AR) are approved by the University Texas Health Science Center at San Antonio (UTHSCSA) Animal Care and Use Committee (IACUC).

All experiments were performed on 8-12-week-old male mice. Rosa26^LSL-tDTomato/+^, TRPV1^cre/+^, vGLUT3^cre/+^, parvalbumin (PV)^cre/+^ and trkB^cre/+-ER^ mouse lines on B6.129 background were obtained from the Jackson Laboratory (Bar Harbor, ME). 5HT3a-GFP and TRPV1-GFP transgenic mouse lines were purchased from the GENSAT program (MMRRC services; UNC, NC and UC Davis, CA, respectively). CGRP^cre/+-ER^ mouse line was kindly provided by Dr. Pao-Tien Chuang (UC San Francisco, San Francisco, CA). MrgA3^cre/+^ and transgenic NPY2R-tdTomato mouse lines were kindly provided by Dr. Xinzhong Dong (John Hopkins University Medical School, Baltimore, MD). MrgD-GFP knock-in mouse line was kindly provided by Dr. Qin Liu (Washington University, St. Louis, MO). trkC^cre/+-ER^ mouse line was generated in Dr. David Ginty’s laboratory (Harvard Medical School, Boston, MA) and kindly provided by Dr. Yu Shin Kim (UTMB, Galveston, TX). In inducible *cre*-carrying mouse lines, apart for trkC^cre/+-ER^, *cre*-recombinase was induced in 6–8 week old mice by three consecutive (every second day) i.p. injection of 100mg/kg tamoxifen (dissolved in corn oil). Cre-recombination in trkC^cre/+-ER^ was induced by gavage administration of tamoxifen (75mg/kg) to pregnant female as described [10]. *Cre*-recombination occurs within 2–3 weeks post-tamoxifen last injection.

### Primary DRG neuronal culture

Reporter mice expressing either GFP or TdTomato gene were deeply anaesthetized with isoflurane (0.3 ml in 1 liter administered for 60–90 sec) and sacrificed by cervical dislocation. L3-L5 DRG were quickly removed, and sensory neurons were cultured as previously described [20]. Briefly, DRG neurons were dissociated by treatment with a 1mg/ml collagenase-dispase (Roche, Indianapolis, IN) solution. Cells were maintained in DMEM supplemented with minimal serum percentage (i.e. 2% fetal bovine serum), 2mM L-glutamine, 100U/ml penicillin and 100μg/ml streptomycin. No growth factor was added to the media. The experiments were performed within 24 h after DRG neuron plating, since electrophysiological profile did not undergo changes within this period.

### Electrophysiology

#### Recording

Recordings were made in whole-cell voltage (holding potential (V_h_) of –60mV) or current clamp configurations at 22–24°C. Data were acquired using an Axopatch 200B amplifier and analyzed with pCLAMP10.2 software (Molecular Devices, Sunnyvale, CA). Recording data were filtered at 0.5–5 kHz and sampled at 2–20 kHz depending on current kinetics. Borosilicate pipettes (Sutter, Novato, CA) were polished to resistances of 2–3 MΩ. If required, access resistance (R_s_) was compensated (40–80%) to the value of 6 MΩ. Data were rejected when R_s_ changed >20% during recording, leak currents were > 100pA, or input resistance was < 100 MΩ. Currents were considered positive when their amplitudes were 5-fold bigger than displayed noise (in root mean square). Standard external solution (SES) contained (in mM): 140 NaCl, 5 KCl, 2 CaCl_2_, 1 MgCl_2_, 10 D-glucose and 10 HEPES, pH 7.4. The standard pipette solution (SIS) contained (in mM): 140 KCl, 1 MgCl_2_, 1 CaCl_2_, 10 EGTA, 10 D-glucose, 10 HEPES, pH 7.3, 2.5 ATP and 0.2 GTP. Drugs were applied by a fast, pressure-driven and computer controlled 4-channel system (ValveLink8; AutoMate Scientific, San Francisco, CA) with quartz application pipettes.

#### Protocols and data analysis

**Fig 1:**
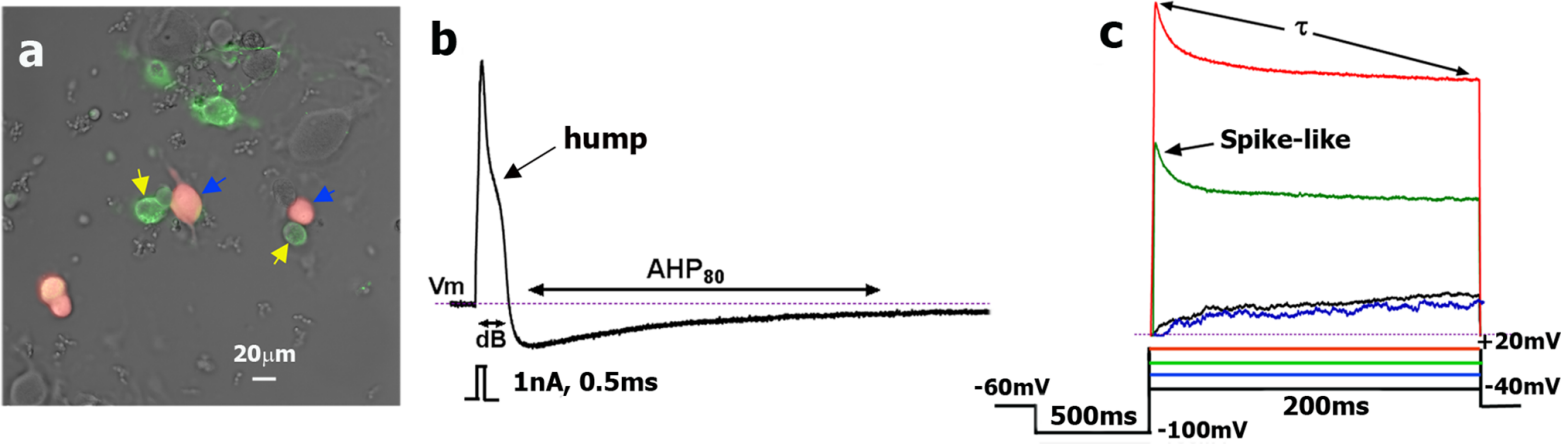
Electrophysiology protocols. **a**. CGRP^+^ neurons (red; marked with blue arrow) selected for recording were classified by size (C_m_ in pF) and staining with IB4 (green; marked with yellow arrow). **b**. Stimulus waveform (1nA, 0.5 msec) indicated below trace generated a single AP in small DRG neuron. We analyzed AP and AHP parameters: resting membrane potential–V_m_; duration at base–dB and the time required for the AHP (measured in mV) to decay by 80%—(AHP_80_). In addition, we measured rise time (RT) and fall time (FT) of AP (Table 1). Characteristic AP “hump” is indicated by black arrow. **c**. Outward current was generated by the indicated waveforms found below traces. The decay constant τ was derived from standard single exponential fits between points indicated by arrows for the final outward current trace (+20 mV). Characteristic “spike-like” peak is shown by arrow on trace generated by stepping to +0 mV.

Before patch clamp recording, DRG cells from reporter mice (single or double heterozygotes) were stained for 0.5–4 h with IB4 Alexa Fluor-488 or IB4 Alexa Fluor-594 (1:1000; Thermo-Fisher Scientific, Waltham, MA). CGRP-cre^+^, Nav1.8-cre^+^ TRPV1-cre^+^, V1-GFP^+^ and other marker-positive DRG neurons were selected randomly for recording. Certain numbers of CGRP-cre^-^, Nav1.8-cre^-^ and other marker-negative DRG neurons were also recorded. Data on CGRP-cre^+^, Nav1.8-cre^+^, TRPV1-cre^+^, V1-GFP^+^ or sensory neuronal marker expression (i.e. red/green or negative), IB4 staining (i.e. positive, weak positive or negative), as well as capacitance (in pF) and resting membrane potential (V_m_ in mV) values were collected prior recording (Fig 1A). On the selected for recording DRG neuron, we used a sequence of protocols: (1) single action potential (AP) in current clamp configuration was generated with 1nA (2nA for >40pF cells) 0.5 msec-current pulse (Fig 1B) [21,22]; (2) after switching to voltage clamp configuration (V_h_ = -60mV), ATP (30μM) current was recording by applying drug for 5 sec; (3) the next protocol in voltage clamp configuration was a step down from V_h_ to -100mV kept for 500ms, and then 200-ms depolarizing command steps (20 mV) were applied from -40mV to a final potential of +20mV (Fig 1C) [21]; and (4) finally, capsaicin (CAP; 100 nM) [23] was applied for 30 sec for recording I_CAP_. In some sets of experiments CAP was substituted with 5-HT (30μM) [24] or mustard oil (MO; 25μM) [25] to evaluate I_5HT_ and MO responses, respectively. Unlike I_5HT_, MO responses were evaluated using Ca^2+^ imaging system. Data was accumulated from 4–7 independent mouse DRG neuronal cultures. Each culture was generated from one mouse and 6 L3-L5 DRG. 16–24 neurons were recorded from each DRG culture.

Cells were considered spherical, and therefore, diameter (d in μm) of cells was calculated from capacitance (C_m_ in pF) values.

d=5x√(Cm/4π)

AP rise time (RT; time from V_m_ to AP peak), fall time (FT; time from AP peak to V_m_ level) and duration at the base (dB; time from V_m_ starting point to V_m_ levels at falling phase of AP) as well as 80% recovery time of after-hyper-polarization to baseline (AHP_80_) were measured from data generated by protocol-1 (Fig 1B). Protocol-2 and -4 revealed algesic responses to ATP, CAP, 5-HT and/or MO as well as I_ATP_ and I_5HT_ characteristics. I_CAP_ and MO-induced response characteristics were not analyzed, since sequential recordings could have desensitized them leading to changes in kinetics and magnitude of these currents/responses. From protocol-3, the trace evoked by +20 mV was fit with a standard (i.e single or double) exponential function.

A1exp[−(t−k)/τ]+C

Fitting and decay tau (τ; ms) calculation was performed using pCLAMP10.2 software (Fig 1C). Presence or absence of “spike-like” feature at steps to 0 and +20 mV was an important clustering variable. An approach for generation of clustering parameters described in details in “Results”; and these parameters outlining 16 sensory neuronal groups are presented in Table 1.

**Table 1:** Sensory neuronal group cluster parameters defined by characterization DRG neurons in marker-reporters.

Group	Size (pF)	dB (ms)	FT:RT	AP shape	AHP80 (ms)	IB4	I_CAP_	I_ATP_	I_5HT_ (pA)	I; τ (ms)	Reporter
S1	<20	>6	>2:1	**hump**	>80	+/-	+	**>500pA** **Act: <1.5 s; Inact: <5 s**	-	-; <15	MrgA3
S2	20–40	>6.5	>2:1	**hump**	>100	-	+	**<300pA** **Act: 2–5 s; Inact: none**	<200	-; <15	5HT3a
S3	10–40	>6	>2:1	**hump**	>50	**+/-**	+	-	<200	-; <15	5HT3a
S4	10–40	>6	>2:1	**bow**	>30	**+**	-	**<300pA** **Act: <1 s; Inact: <3 s**	<200	-; <15	MrgD
S5	20–40	>6	>2:1	**bow**	10–30	**+**	-	-	-	-; <15	MrgD
S6	<20	2.5–5.5	>1:1	**-**	10–60	**-**	**+**	-	-	-; 10–30	V1-GFP
S7	<20	>4	>1.5:1	**deflection**	30–100	**-**	-	-	-	-; 10–30	vGLUT3
M1	>35	4–6.5	>2:1	**hump**	>60	-	-	-	**>600**	**+; 10–40**	5HT3a
M1a	>35	4–6.5	>2:1	**hump**	>60	-	-	<500pA Act: <1.5 s; Inact: 15–30 s	**>600**	**+; 10–40**	5HT3a
M2	>30	2.5–4	**<1:1**	**-**	<10	-	-	-	-	**-; >40**	trkB
M3	>50	3–4.5	>1:1	**deflection**	>80	-	-	-	<400	**+; 10–40**	NPY2R
M3a	>50	3–4.5	>1:1	**deflection**	>60	-	-	<500pA Act: >1.5 s; Inact: 15-30s	<400	**+; 10–40**	NPY2R
M4	>60	1.5–3	**<1:1**	**-**	**>70**	-	-	-	-	**+; <20**	trkC
M5	>60	1–2.5	**<1:1**	**-**	**15–35**	-	-	<500pA Act: >1.5 s; Inact: >20 s	**<300**	**+; <20**	trkC PV
M6	>60	1–2	**<1:1**	**-**	**5–25**	-	-	<500pA Act: >1.5 s; Inact: >20 s	**-**	**-; >40**	trkC^-^
M7	>40	1–2	**<1:1**	**-**	**3–15**	-	-	-	**-**	**-; >40**	PV

Groups in bold, sub-groups in italic; Peptidergic sensory neuronal groups are highlighted in yellow and non-nociceptive groups (LTMR) are highlighted in blue. Non-peptidergic nociceptors are unmarked. Mandatory parameters are in bold.

I_ATP_—current size and kinetic parameters are noted. *Act* is time to reach 95% of peak. Inact is time for 50% decline during drug delivery.

IB4—week expression is marked “+/-“;trkC^-^ is trkC negative subset of medium-large-sized neurons

Groups with clearly detected “spike-like” feature for outward current (I) is marked “+”; groups with no “spike-like” feature are marked “-“. The τ obtained after fitting with single or double exponential equation is noted.

**Fig 2:**
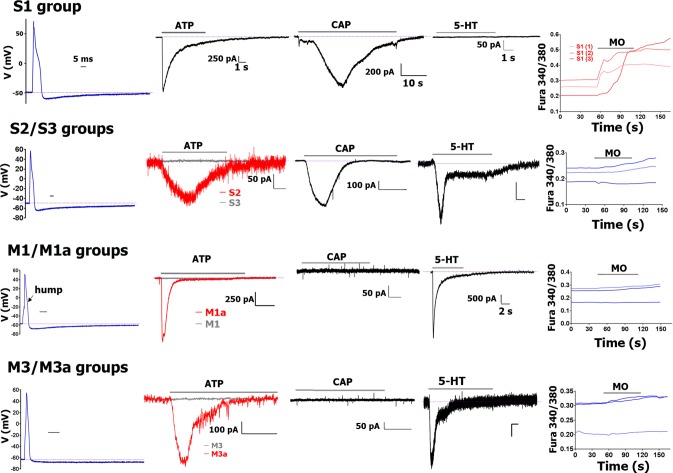
AP traces and algesic response profile for sub-classes of CGRP-cre^+^ DRG neurons. For each CGRP-cre^+^ sensory neuronal group recorded from mouse DRG, the AP, response to ATP (30μM), capsaicin (100nM; CAP), 5-HT (30μM) and mustard oil (10μM; MO) are presented from left to right. The AP time scale (horizontal bar) is 5 msec for each panel. I_ATP_ time scale is 1 sec for each panel. I_ATP_ magnitude (vertical bar) scales are indicated for each panel. Name of the groups are also indicated when two I_ATP_ traces are presented on a panel. I_CAP_ time scale is 10 sec for each panel. I_CAP_ magnitude scale is indicated. I_5-HT_ was recorded from S3, but not S2 neurons. I_5-HT_ time and magnitude scales are 1 sec and 50 pA, respectively, for each panel. An exception is the M1/M1a group that has a large I_5-HT_ current. MO responses were measured by Ca^2+^ imaging. CGRP-cre^+^ sensory neuronal groups are shown for each row. Drug application times are illustrated by horizontal bar above traces. More complete information on subgroups is presented in Table 2.

Characteristic features of AP waveform on its return to baseline, such as “hump”, “bow” and “deflection” (a.k.a. lesser pronounced “hump”) are noted, and are shown on Figs 1A, 2 and 4A.

### Ca^2+^ imaging

MO responses were acquired with fluorescent Ca^2+^ imaging as previously described [26]. Data were collected and analyzed with NIS-elements software (Nikon Instruments, Melville, NY). The experiments were performed in SES solution using calcium-sensitive, cell permeable dye Fura-2 AM (2 μM; Molecular Probes, Carlsbad, CA). Mean value of the basal level of intracellular Ca^2+^ ([Ca^+2^]_i_) was collected for 60 s prior to agonist applications. The net changes in Ca^+2^ influx were calculated by subtracting the basal level of [Ca^+2^]_i_ from the peak [Ca^+2^]_i_ value achieved after exposure to the agonists. Ca^2+^ imaging experiments were combined with patch-clamp recording.

### Immunohistochemistry (IHC)

L3-L5 DRG were dissected from CGRP^cre-ER/-^;Rosa26^LSL-tDTomato/-^ (i.e. double heterozygotes) and Nav1.8^cre/-^;Rosa26^LSL-tDTomato/-^ 4% paraformaldehyde-perfused mice. Tissues were additionally fixed with 4% paraformaldehyde for 15 min, cryo-protected overnight with 30% sucrose in phosphate buffer, embedded in Neg 50 (Richard Allan Scientific, Kalamazoo, MI); and 30μm cryo-sections were generated as previously described [20]. IHC was carried out as previously described [20]. IHC was simultaneously performed on 6–12 sections generated from 3 animals. The following previously characterized primary antibodies were used: anti-TRPV1 guinea pig polyclonal (Neuromics; Bloomington, MN; catalogue GP14100; 1:700) [27]; anti-CGRP rabbit polyclonal (Sigma; C8198; 1:300) [28,29,30]; anti-tyrosine hydroxylase (TH) rabbit polyclonal (Pel-Freez; Rogers, AR; P40101; 1:400) [31,32]; anti-mrgD rabbit polyclonal (Alamone Lab; AMR-061; 1:200) [33]; anti-NPY2R rabbit polyclonal (Sigma; SAB4502029; 1:50; evaluated in DRG from NPY2R-TdTomato reporter); anti-5HT3a rabbit polyclonal (Alomone Lab; ASR-031; 1:100; evaluated in DRG from 5HT3a-GFP reporter) [34]; anti-trkC goat polyclonal (R&D systems; Minneapolis, MN; AF1404; 1:200) [5,35]; anti-trkB goat polyclonal (R&D systems; AF1494; 1:200) [5,36]; rabbit anti-parvalbumin (Swant, PV25, 1:500) [5,37,38]; and rabbit anti-Calbindin D28k (Swant, CB-38a, 1:500) [5,37]. Antibodies for mrgD, 5HT3a and NPY2R produced a much weaker signal than TdTomato reporters. Therefore, signal to noise (i.e. background) ratio is low. For these antibodies, we adjusted (i.e. increased) their intensity and reduced background (i.e. contrast). Sections were incubated with species appropriate Alexa Fluor secondary antibodies (1:200; Molecular Probes, Eugene, OR). Images were acquired using a Nikon Eclipse 90i microscope (Melville, NY, USA) equipped with a C1si laser scanning confocal imaging system. Images were processed with NIS-elements software (Nikon Instruments, Melville, NY).

### Statistics

Control IHC was performed on tissue sections processed as described but either lacking primary antibodies or lacking primary and secondary antibodies. Cell counts from IHC images acquired as Z-stuck were performed using Image J software. Total cells/section and cells positive for each marker as well as the combinations of markers were counted. Intensity of immunoreactivity or TdTomato labeling was also calculated with Image J software; subtractions of background intensity from signal levels were applied. We used 3 independent mice to generate sections and counted 3–5 sections per mouse. Mean values from n = 3–5 were generated per animal, and standard error of the mean was calculated on this basis. Data were presented on scatter plots with bars.

GraphPad Prism 7.0 (GraphPad, La Jolla, CA) was used for statistical analyses. Data in the figures are mean ± standard error of the mean (SEM), with “n” referring to the number of recorded and analyzed cells. Differences between electrophysiologically characterized groups were assessed by unpaired *t*-test or regular 1-way ANOVA with Tukey’s post-hoc tests, each column was compared to all other columns. A difference is accepted as statistically significant when p<0.05. Interaction F ratios, and the associated p values are reported.

## Results

### Approaches to electrophysiological classification of sensory neuronal groups

Depending on the recording technique/approach, there are many accepted ways to classify sensory neurons [39]. One traditional classification is based on myelination status and fiber conduction velocities, which can be measured from recordings from *ex vivo* and *in vivo* preparations [40]. Each of these groups (i.e. un-myelinated and myelinated), in turn, forms a heterogeneous population of sensory neurons that have various functions and contain different fiber subtypes [4,22,41]. In addition, sensory neuronal clusters differ in their innervation target [42,43]. This type of classification of sensory neurons requires comprehensive *in vivo* extracellular or, preferably, intracellular recording and application of physiological stimuli to innervation sites [22,42,44]. Alternative approaches are the use of patch-clamp recording and classification according to AP properties, sensitivity to algesic agents and appearances of a variety of voltage-gated currents [21,45]. These approaches could become especially powerful after detailed characterization of sensory neuronal groups expressing defined markers [5,6,9,46]. The use of information from next generation sequencing for transcriptional profiling of different types of sensory neurons [1,12] would provide additional important details regarding patch-clamp recorded subsets.

**Fig 3:**
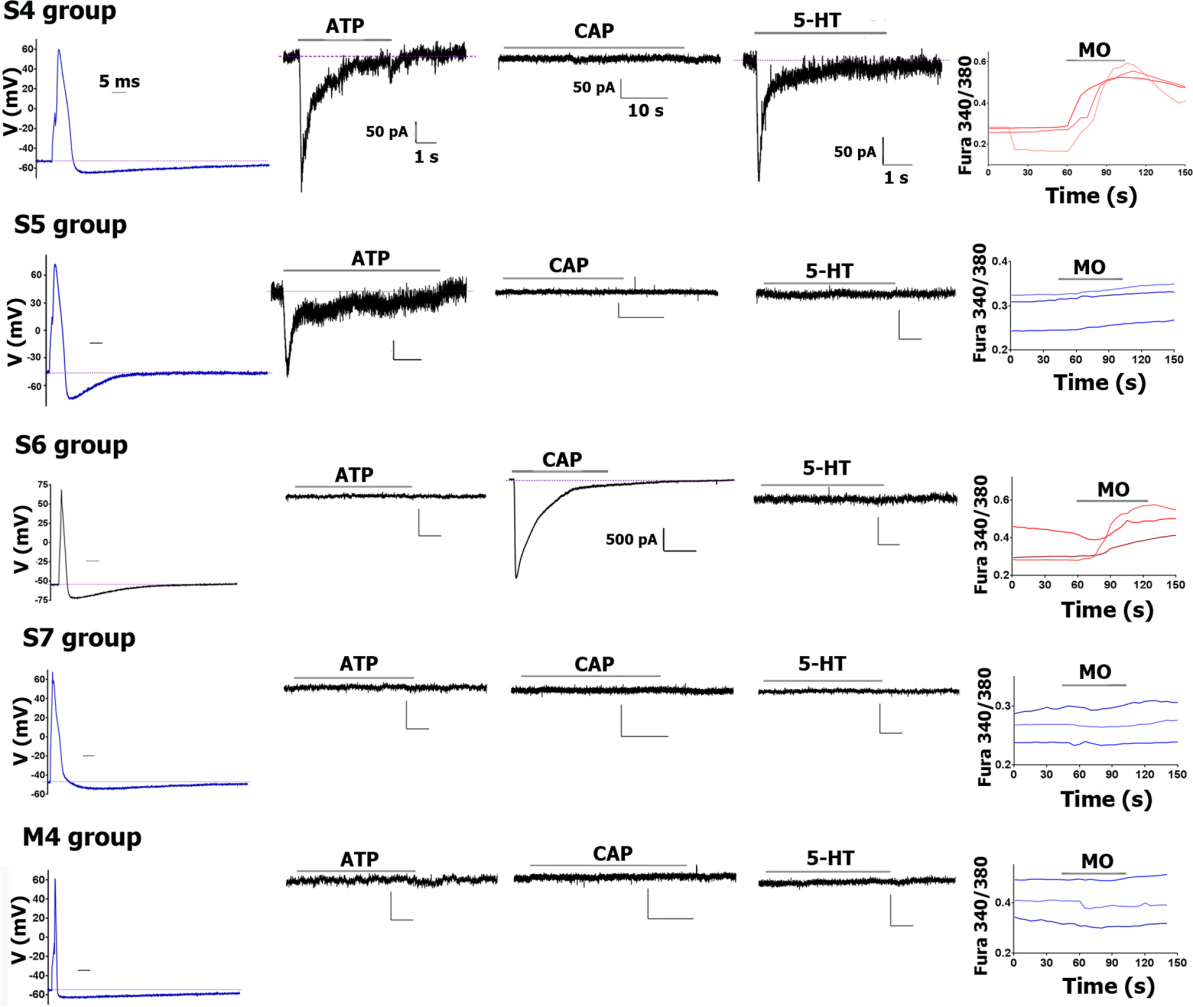
AP traces and algesic response profiles for sub-classes of Nav1.8-cre^+^/CGRP-cre^-^ DRG neurons. For each Nav1.8-cre^+^/CGRP-cre^-^ sensory neuronal group recorded from mouse DRG, the AP, response to ATP (30μM), CAP (100nM), 5-HT (30μM) and MO (10μM) are presented from left to right. The AP time scale (horizontal bar) is 5 ms for each panel. I_ATP_ time and magnitude (vertical bar) scales are 1 s and 50 pA, respectively, for each panel. I_CAP_ time scale is indicated, and magnitude scale is 50pA for each panel, except for S6 neuronal group. I_5-HT_ time and magnitude scales are 1 s and 50 pA, respectively, for each panel. MO responses were measured by Ca^2+^ imaging. Names of Nav1.8-cre^+^/CGRP-cre^-^ sensory neuronal groups are indicated. Drug application times are illustrated by horizontal bar above traces. More complete information on subgroups is presented in Table 3.

**Fig 4:**
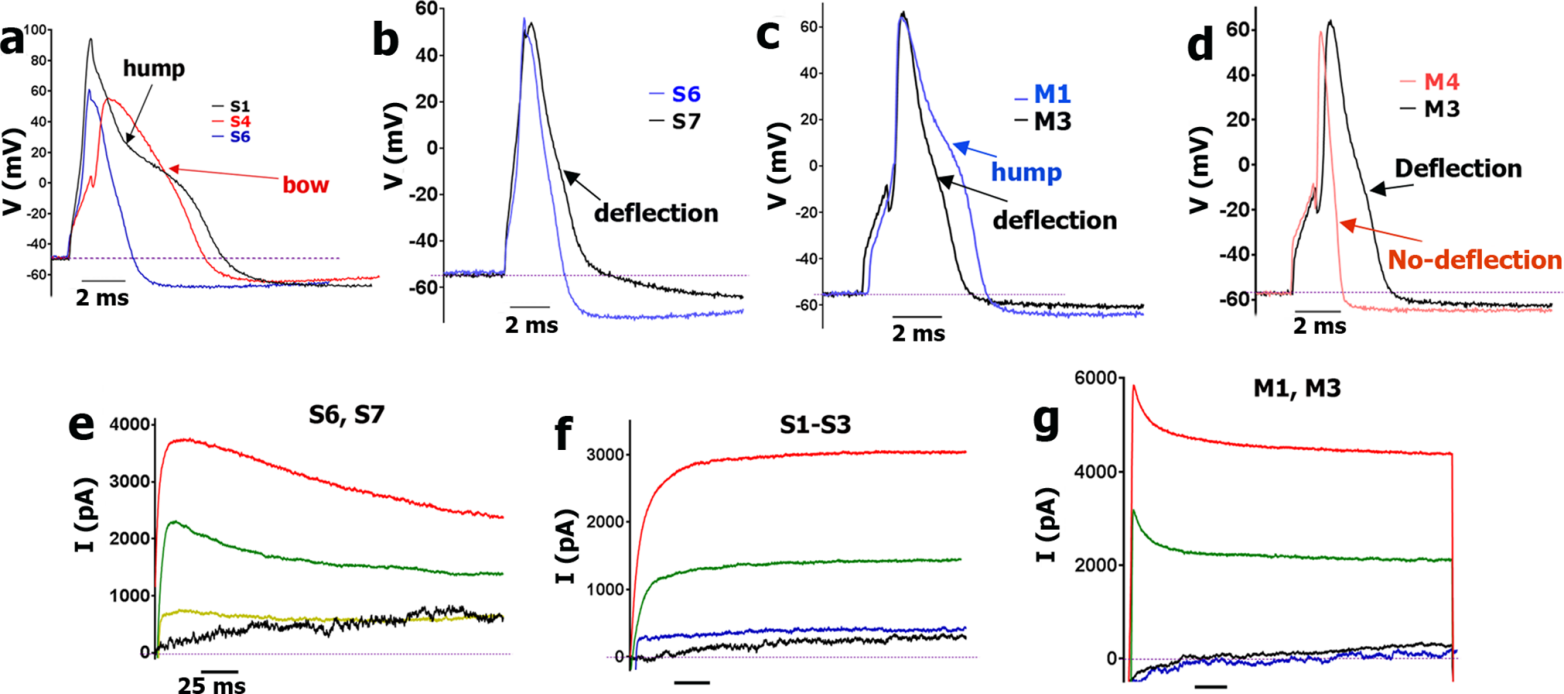
AP differences between sensory neuronal groups, and outward currents in Nav1.8-cre^+^ neurons. **a**. Comparison of AP shapes generated in S1 CGRP-cre^+^/Nav1.8-cre^+^, S4 CGRP-cre^-^/Nav1.8-cre^+^ and S6 CGRP-cre^-^/Nav1.8-cre^+^ neurons. AP “hump” in S1 neurons and “bow” in S4 neurons are indicated with black and red arrows, respectively. S6 neurons’ AP does not display any deflection during the falling phase of AP. **b**. Comparison of AP in S6 and S7 neurons. Deflection on the falling phase of S7 neuron AP is indicated by black arrow. **c**. Comparison of single AP in M1 and M3 CGRP-cre^+^ neuronal group. “Hump” is marked with blue arrow, while “deflection” is indicated with black arrow. **d**. Comparison of AP in M3 and M4 Nav1.8-cre^+^/CGRP-cre^-^ DRG neurons. M3 neuron’s AP “deflection” is indicated with black arrow, and M4 neuron’s AP with no “deflection” is shown with red arrow. **e**. Typical outward current (I) produced from Nav1.8-cre^+^/CGRP-cre^-^ S6 and S7 neuronal groups. **f**. Typical I produced from the CGRP-cre^+^ S1-S3 group neurons. **g**. Typical I produced from CGRP-cre^+^ M1 and M3 group neurons. Names of neuronal groups are specified above traces. The time scale (horizontal bar) is 25 ms for each panel.

**Fig 5:**
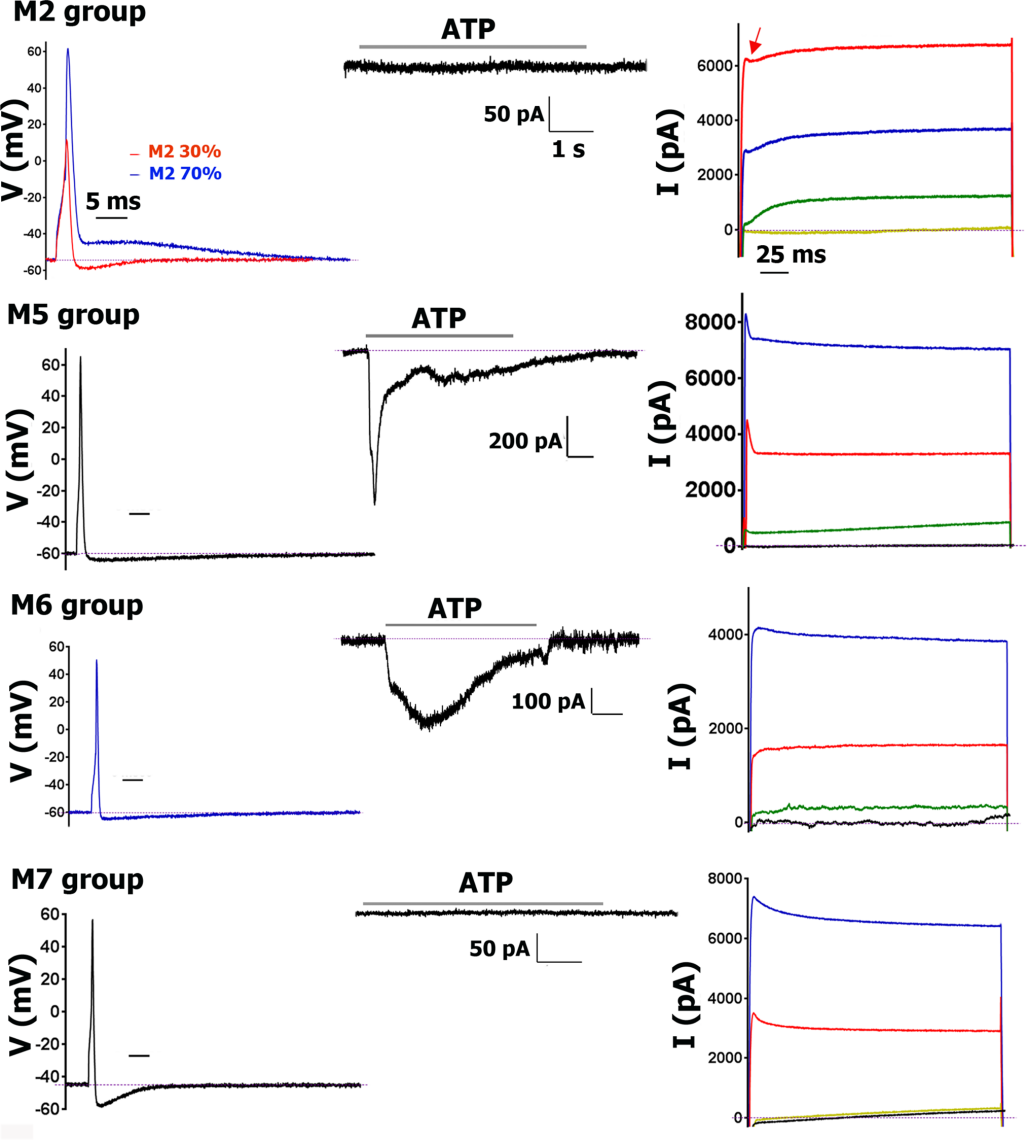
AP traces and algesic response profiles for sub-classes of Nav1.8-cre^-^ DRG neurons. For each Nav1.8-cre-negative (Nav1.8-cre^-^) sensory neuronal group recorded from mouse DRG, the AP, response to ATP (30μM) and outward current (I; see “Method” protocol-3) are presented from left to right. “Partially developed” small spike-like feature in the M2 Nav1.8-cre^-^ neurons is indicated with a red arrow. The AP time scale (horizontal bar) is 5 ms for each panel. I_ATP_ time scale is 1 s for each panel; and magnitude scales (vertical bars) are indicated separately for each panel. I current time scale is 25 ms for each panel. Names of Nav1.8^-^ sensory neuronal groups are indicated. Drug application times are illustrated by horizontal bar above traces. More complete information on subgroups is presented in Tables 3 and 5.

Patch clamp recording from selected DRG neurons yielded data on 13 variables: cell size, dB, FT:RT ratio, AP kinetics, characteristic features of AP shapes, AHP_80_, IB4 staining, responsiveness to 5-HT, ATP, MO and CAP, τ (tau) from fitting of voltage-gated current, and presence or absence of a “spike-like” feature on outward portions of voltage-gated currents (Table 1). IB4 signals were reported as non-detectable, weak or strong with clear plasma membrane staining [47]. Strongly stained IB4^+^ neurons have been classically considered non-peptidergic [48], while some of weakly stained IB4^+^ neurons (marked as +/- in Tables 1–5) are peptidergic [47]. ATP responses were classified according to their magnitude and kinetics. CAP, 5-HT and MO responses was qualified as positive (>50 pA) or negative. “Spike-like” feature was revealed by the presence of a sharp peak on outward portion of current generated by stepping to 0 and +20mV (Fig 1C). It was noted that some myelinated A-fiber produce a “spike-like” feature (Fig 1C) [21,45]. It worth to note that “spike-like” feature is likely not representative of only A-current as it was previously interpreted [21], but rather a depolarizing voltage-clamp error resulting from the proceeding of the large inward sodium current. Nevertheless, “spike-like” feature can be used as a clustering variable to distinguish some of A-fiber containing neurons [21]. In some neuronal groups, shape of outward current was biphasic (see Table 1). Before analyzing data from CGRP-cre^+^, Nav1.8-cre^+^, TRPV1-cre^+^ and V1-GFP^+^ neurons and assigning them to particular sensory neuronal groups, parameters for definitive and unambiguous clustering were created on basis of recording of DRG neurons from reporter mice, such as mrgD-GFP^+^, trkC-cre^+^, trkB-cre^+^, 5HT3a-GFP^+^, NPY2R^+^, PV-cre^+^ and vGLUT3-cre^+^ (Table 1). Mandatory variables (in bold) are essential for getting clear “separation” between some of sensory neuronal groups (Table 1). Clustering parameters generated on the basis of recording of sensory neurons from reporter mice allows clearer separation between groups to an extent that assigning a recorded neuron to particular group could be performed without software. Nevertheless, cluster analysis based on a distribution model (XLStat and NCSS) confirmed findings [21]. Analysis of recordings from >600 DRG neurons expressing or not expressing various markers (including CGRP-cre^+^, Nav1.8-cre^+^, TRPV1-cre^+^ and V1-GFP^+^) revealed 7 groups of small neurons (<35pF) and 7 groups of medium-large neurons (>35pF) (Table 1; Figs 2, 3, 4 and 5). Two groups M1 and M3 contain sub-groups, M1a and M3a respectively. These sub-groups were only distinguished by responsiveness to ATP. Each neuron can be assigned to one of these groups based on the criteria that every parameter, with the possibility of one exception, should fit the characteristics summarized in Table 1. Moreover, variables in bold font were a mandatory fit for specified neuronal groups (Table 1). If neuron could not be assigned to either group, it was discarded from further analysis. Only 14 neurons from >600 recorded did not fit any group outlined in Table 1.

**Table 2:** Properties of CGRP^cre-ER^/TdTomato^+^ sensory neuronal groups.

Group	N	Vm	Size (pF)	dB (ms)	AHP80 (ms)	τ(ms)	I_ATP_ (pA)	IB4	CAP	MO	5-HT	Marker reporters
**S1**	12	-51.7±1.8	15.8±0.9	8.8±0.3	176.1±22.9	7.1±0.8	1086±215	+/-	+	+	-	mrgA3, TRPV1
**S2**	7	-48.6±1.5	27.4±2.3*	8.2±0.6	138.9±30.4	8.8±2.3	118.8±18**	-	+	-	+/-	TRPV1
**S3**	23	-48.1±1.1	17.1±1.4	7.6±0.3*	143.4±12.9	6.5±0.7	-	+/-	+	-	+/-	TRPV1, 5HT3a
**M1**	15	-53.0±0.8	40.8±3.0****	5.0±0.3****	160.0±24.5	25.2±3.6***	-	-	-	-	+	5HT3a
*M1a*	5	-55.8±0.5	44.8±3.8****	5.2±0.4****	126.4±22.1	29.2±3.3***	585±317	-	-	-	+	5HT3a
**M3**	7	-56.3±0.9	57.0±5.4****	3.6±0.1****	120.7±12.8	34.2±7.3****	-	-	-	-	+/-	NPY2R
*M3a*	5	-56.2±1.9	57.4±7.9****	3.7±0.2****	171.2±38.1	36.1±6.3****	458±158	-	-	-	+/-	NPY2R

5-HT–large and fast 5-HT current is only noted in M1/M1a. 5-HT responses are small in S2, S3, M3 and M3a groups, and are marked as “+/-“. Weak staining with IB-4 is marked as “+/-“. Statistic is 1-way ANOVA, control column is S1, post-hoc analysis Bonferroni

*p<0.05

**p<0.01

***p<0.001

****p<0.0001. If difference is insignificant (P>0.05), then no sign is shown.

**Table 3:** Properties of Nav1.8^cre^/TdTomato^+^ sensory neuronal groups.

Group	N	Vm	Size (pF)	dB (ms)	AHP80 (ms)	τ (ms)	I_ATP_ (pA)	IB4	CAP	MO	5-HT	Marker reporters
S1	9	-50.3±1.8	15.8±0.8	8.8±0.3	176.1±22.9	5.5±0.8	1224±237	+/-	+	+	-	mrgA3, TRPV1
S2	5	-49.2±2.8	26.3±3.5*	8.2±0.6	138.9±30.4	9.2±2.0**	130±32****	-	+	-	+/-	TRPV1
S3	15	-47.6±1.2	18.4±1.6	7.6±0.3	143.4±12.9	6.1±0.2	-	+/-	+	-	+/-	TRPV1, 5HT3a
**S4**	12	-46.3±1.5	27.2±2.2*	7.3±0.3*	76.3±9.5	10.9±2.5**	246±51****	+	-	+	+/-	mrgD, 5HT3a
**S5**	11	-41.3±2.4	25.1±1.3*	7.6±0.4	19.6±3.8	4.5±0.6	-	+	-	-	-	mrgD
**S6**	8	-52.4±2.3	13.6±0.8	3.7±0.3****	34.2±12.5	16.5±1.3***	-	-	+	+	-	TRPV1
**S7**	6	-50.2±3.8	15.2±1.7	5.6±0.5***	81.7±21.0	18.1±3.6**	-	-	-	-	-	TH, vGLUT3
M1	11	-54.1±0.9	43.2±2.5****	5.2±0.4****	144.2±15.4	20.4±1.4***	-	-	-	-	+	5HT3a
M1a	4	-55.3±2.7	46.3±4.3***	4.9±0.5****	101.1±42.2	15.2±5.3**	311±144**	-	-	-	+	5HT3a
M3	9	-56.0±1.6	62.3±5.6****	3.8±0.2****	132.3±18.8	38.7±8****	-	-	-	-	+/-	NPY2R
M3a	3	-59.2±4.6	60.1±8.4***	3.7±0.4****	163.2±38.3	29.2±5.4***	435±239*	-	-	-	+/-	NPY2R
**M4**	6	-57.8±3.7	82.2±8.7****	2.4±0.2****	121.2±24.2	7.5±2.4	-	-	-	-	-	trkC

CGRP-cre-ER/TdTomato^+^ groups are highlighted by yellow in the “Group” column and TRPV1-cre/TdTomato groups are highlighted by blue in the “N” column. 5-HT–large and fast 5-HT current is only noted in M1/M1a. 5-HT responses are small in S2, S3, M3 and M3a groups, and are marked as “+/-“. MO responses are largest in S4 group, and are almost undistinguishable from current noise in S7 group. IB-4 staining in group S1 and S3 is weak; and are marked “+/-“. Statistic is 1-way ANOVA, control column is S1, post-hoc analysis Bonferroni

*p<0.05

**p<0.01

***p<0.001

****p<0.0001. If difference is insignificant (P>0.05), then no sign is shown.

**Table 4:** Properties of TRPV1-GFP^+^ sensory neuronal groups.

Group	N	Vm	Size (pF)	dB (ms)	AHP80 (ms)	τ (ms)	I_ATP_ (pA)	IB4	CAP	MO	Marker reporters
S1	10	-49.3±2.5	14.4±0.7	6.7±0.3	234.6±31.5	4.0±0.5	1103±180	+/-	+	+	mrgA3
S2	7	-49.4±2.7	27.9±1.3****	8.1±0.5	196.7±23.7	11.8±2.2**	223±31**	-	+	-	5HT3a
S3	19	-48.6±2.1	21.2±1.5**	8.5±0.5*	151.8±18.7	6.2±0.6	-	+/-	+	-	5HT3a
**S6**	11	-47.7±3.8	12.5±0.9	4.1±0.3**	45.2±17.5***	15.8±3.5****	-	-	+	+	?

CGRP-cre-ER/TdTomato^+^ groups are highlighted by yellow in “Group” column. Sign “?” marks unknown or a candidate marker. Thus, S6 group marker could be somatostatin (SST). IB-4 staining in group S1 and S3 is weak, and marked “+/-“. Statistic is 1-way ANOVA, control column is S1, post-hoc analysis Bonferroni

*p<0.05

**p<0.01

***p<0.001

****p<0.0001. If difference is insignificant (P>0.05), then no sign is shown.

**Table 5:** Properties of marker-defined groups of sensory neuros.

Group	N	Vm	Size (pF)	dB (ms)	AHP80 (ms)	τ (ms)	I_ATP_ (pA)	IB4	5HT (pA)	CAP	MO	Tr. Gr.
**MrgprA3/TdTomato+**												
**S1**	19	-51.4±0.92	15.7±1.36	7.48±0.4	170.8±18.5	6.02±0.37	1438±162	+/-	-	+	+	NP-2
**MrgprD-GFP+**												
**S4**	8	-45.1±1.46	26.1±3.1	7.6±0.4	82.3±13.8	11.4±3.67	+	+	153.1±33.7	-	+	NP-1
**S5**	9	-40.3±3.21	27.4±2.0	7.8±0.4	22.2±4.3	5.2±1.36	-	+	-	-	-	NP-1
**vGLUT3/TdTomato+**												
**S7**	21	-51.8±1.63	15.0±1.7	5.15±0.3	71.2±11.2	15.3±1.34	-	-	-	-	-	TH
**5HT3a-GFP+**												
**S2**	10	-47.7±1.23	21.8±1.8	7.27±0.5	172.6±25.4	9.2±1.5	+/-	-	75.3±16.2	+	-	PEP-1
**S3**	5	-46.8±1.49	25.0±2.2	8.70±1.0	141.7±12.6	7.1±1.2	+	+/-	150.6±42.6	+	-	PEP-1
**M1**	18	-51.4±0.71	41.4±2.3	5.13±0.3	124.5±13.7	28.8±7.1	-	-	1937±135	-	-	PEP-2
*M1a*	3	-52.4±2.88	47.0±3.5	4.51±0.7	95.1±42.5	21.2±9.4	-	-	2523±453	-	-	PEP-2
**trkB/TdTomato+**												
**M2**	22	-54.7±0.48	38.6±2.44	3.2±0.1	N/A	68.2±10.6	-	-	-	-	-	NF-1
**NPY2R-TdTomato+**												
**M3**	16	-55.1±0.58	65.3±3.8	3.52±0.1	111.8±5.9	33.2±4.2	-	-	441.5±52.1	-	-	PEP-2
*M3a*	5	-56.8±1.73	72.2±9.1	3.42±0.4	99.4±21.2	40.6±5.8	-	-	335.2±28.9	-	-	PEP-2
**trkC/TdTomato+**												
**M4**	12	-59.8±1.46	92.8±8.1	2.4±0.2	153.5±18.5	8.4±1.23	-	-	-	-	-	NF-3
**M5**	14	-58.3±1.25	81±5.76	1.7±0.1	17.2±2.5	11.2±2.27	457±88.7	-	122.4±41.2	-	-	NF-4 or 5
**PV/TdTomato+**												
**M5**	6	-57.5±1.08	90.0±7.12	1.9±0.3	21.7±2.1	9.8±3.23	318±202.7	-	122.4±41.2	-	-	NF-4 or 5
**M7**	31	-54.5±0.90	72.4±3.04	1.6±0.1	8.4±0.73	56.9±2.35	-	-	-	-	-	NF-4 or 5

*Tr*.*Gr*. represent putative match between groups characterized here by electrophysiology and IHC with defined sensory neuronal markers and groups defined by single-cell sequencing (see Fig 4 in Usoskin et al., 2015).

We did not evaluate mouse reporters that represent S6 (i.e. CGRP^-^/TRPV1^+^) and M6 groups. Therefore, these two groups are missing in this table.

S6 could match NP-3 (Usoskin et al., 2015); and M6 could match NF-2, which has a main marker calbindin-28kDa (Usoskin et al., 2015).

*PV* is parvalbumin.

5 NPY2R-TdTomato^+^ neurons did not fit any group outline in this table.

### CGRP^cre-ER^/TdTomato expressing sensory neuronal groups

Seventy seven CGRP^cre-ER^/TdTomato^+^ (i.e. CGRP-cre^+^) either IB4^+^ or IB4^-^ DRG neurons (Fig 1A) were recorded with sequential protocols as described in the “Methods”. All but 3 CGRP-cre^+^ neurons could be assigned to one of 5 main clusters (in bold) and two sub-clusters (in italic; Table 2).

#### Small-sized (<35pF) CGRP-cre^+^ DRG neurons

CGRP-cre^+^ DRG neurons classified as S1 had weak IB4 staining, broad AP (i.e. high dB values) with a “hump” and slow AHP (Fig 2; Table 2). The most distinct feature of S1 neurons, which was not encountered among any other neuronal groups, is a large I_ATP_ (>500pA) with fast (<1.5 sec) activation and slow (<5 sec) inactivation kinetics (see Table 1; Fig 2). S1 CGRP-cre^+^ neurons responded to CAP and MO, but not 5-HT (Fig 2). Outward current (I) from S1 neurons did not have “spike-like” peak (Fig 4F); and has low τ (<15 msec; Table 1). Analysis of recording from neurons expressing various reporter-markers revealed that the S1 group closely matched a sensory neuronal cluster expressing MrgprA3^cre^/TdTomato (Table 5).

AP and AHP characteristic and shape, as well as I features of S2 and S3 CGRP-cre^+^ DRG neurons were similar to those of S1 group neurons (Figs 2 and 4F; Tables 1 and 2). S2 and S3 neurons are responsive to CAP, but not MO (Fig 2). S2, unlike S3 CGRP^+^ neurons had no IB4 staining. S2 and S3 are also distinct in responses to ATP: S2 had slow small I_ATP_ (Fig 2; Table 2), while S3 was not gated by ATP. S2 did not have a distinct marker among those analyzed (Table 5). However, S3 neurons were weakly 5HT3a-GFP^+^ (Table 5). Hence, tiny I_5HT_ was recorded from S3, but not S2 neurons (Fig 2).

#### Medium-large-sized (>35pF) CGRP-cre^+^ DRG neurons

CGRP-cre^+^ DRG medium-large neurons belong to two main groups: M1/M1a and M3/M3a (Table 2). Unlike M1 and M3, M1a and M3a had fast and medium-to-large sized I_ATP_ (Fig 2; Tables 1 and 2). CGRP-cre^+^ M1/M1a and M3/M3a neurons had no IB4 staining, are insensitive to CAP and MO (Fig 2), and their outward currents have a pronounced “spike-like” feature. M1/M1a and M3/M3a τ values are higher than in S1-S3 group neurons (Fig 4G; Tables 1 and 2). M1/M1a AP dB is faster than S1-S3 dB values (1-way ANOVA; F (6, 56) = 24.43; P<0.0001), but a slight “hump” on AP down stroke phase is clearly detectable (Fig 2; Table 2*)*. One of main distinctions M3/M3a from M1/M1a is that M3/M3a dB is faster (1-way ANOVA; F (3, 20) = 6.835; P = 0.0024; Table 2); and a “hump” is not pronounced and appears as a “deflection” (Fig 4C*)*. Both M1/M1a and M3/M3a have slow AHP (Table 2). Recording from marker-expressing neuronal subsets showed that M1/M1a strongly expresses 5HT3a-GFP, while M3/M3a is weakly 5HT3a-GFP^+^ (Table 5). As a result, M1/M1a has a very large I_5HT_ (1–4 nA, while I_5HT_ in M3/M3a is <0.4 nA; Fig 2). The distinct marker for M3/M3a neurons is NPY2R (Tables 1, 2 and 5). Weak 5HT3a expression was detected in NPY2R^+^ DRG neurons isolated from double reporter NPY2R-TdT/5HT3a-GFP mice. It could be noted that average-sized responses to 5-HT were recorded only from ≈40% of NPY2R^+^ neurons (Tables 2, 3 and 5). Altogether, M1/M1a represents 5HT3a^+^/NPY2R^-^ neurons, and M3/M3a is NPY2R^+^ neurons.

### Nav1.8^cre^/TdTomato expressing sensory neuronal groups

One hundred and three Nav1.8^cre^/TdTomato (Nav1.8-cre^+^) DRG neurons, which are IB4^+^ or IB4^-^, were recorded with a sequence of protocols as described in “Methods”. All but 4 Nav1.8-cre^+^ neurons were assigned to one of 10 main clusters (in bold) and two sub-clusters (in italic; Table 3). Every CGRP-cre^+^ neuronal group (S1-S3, M1/M1a and M3/M3a) was found to be Nav1.8-cre^+^ (Tables 2 and 3). Hence, only Nav1.8-cre^+^/CGRP-cre^-^ groups are described below.

#### Small-sized (<35pF) Nav1.8-cre^+^/CGRP-cre^-^ DRG neurons

S4 and S5 neurons had strong staining with IB4, and did not respond to CAP (Table 3). They also displayed a unique slow AP (i.e. high dB values) shape with “bow” during AP fall phase (Figs 3 and 4A). AHP was substantially faster in the S5 than S4 group (for S5 20.4±0.7 for S4 89.3±7.8; *t*-test; t = 6.5 df = 14; p<0.0001; n = 8–12; Table 3; Fig 3). Other distinctions between S4 and S5 were responses to ATP, MO and 5-HT. S4, but not S5 neurons, responded to all of these algesic agents (Table 5; Fig 3). Outward current shape and τ were similar between S1-S3 and S4-S5 groups (Table 3). Analysis of recording from neurons expressing various reporter-markers revealed that S4 and S5 groups match clusters expressing MrgprD-GFP (Table 5).

Neurons of S6 and S7 groups were smallest sizes (i.e. <20pF), and not stained by IB4, nor do they respond to ATP or 5-HT (Table 3; Fig 3). Distinctively, S6 and S7 neurons have faster dB than other small neurons of S1-S5 groups (1-way ANOVA; F (6, 56) = 24.4; P<0.0001; Table 3; Fig 3). S6 and S7 were also unlike S1-S5 in regards to outward currents (I). Thus, I of S6 and S7 have higher than S1-S5 τ values (Table 3; Fig 4E vs Fig 4F). Unlike S6, neurons of the S7 group exhibited a slight deflection on the falling portion of AP (Fig 4B). AHP was relatively faster (compared to S1-S3) in S7 and especially S6 neurons (1-way ANOVA; F (2, 54) = 18.2; P<0.0001; Table 3; Figs 2 and 3). However, AP and AHP characteristics are not reliable in assigning neurons to S6 or S7 group. The definitive feature of S6 neurons that separates them from S7 was their CAP and MO sensitivity (Table 3; Fig 3). Moreover, I_CAP_ in S6 neurons were largest compared to other TRPV1-GFP positive neuronal groups (Table 4; Figs 2 and 3). Properties of S7 neurons were matched to characteristics of vGLUT3-cre^+^ DRG neurons from vGLUT3^cre^/TdTomato mice (Table 5). It could be noted that vGLUT3-cre^+^ neurons are completely CAP insensitive. It is also worth noting that ≈25% of vGLUT3-cre^+^ neurons have C_m_ of >50 pF, and their electrophysiological profiles distinct them from S7 neurons. These larger vGLUT3-cre^+^ could be vGLUT3^-^ in the adult due to a fate map [49]. Data from larger vGLUT3-cre^+^ neurons were not included in Table 5.

#### Medium-large-sized (>35pF) Nav1.8-cre^+^/CGRP-cre^-^ DRG neurons

All but one medium-large Nav1.8-cre^+^ neuronal group co-expressed CGRP-cre^+^ (Table 3*)*. An exception was group M4. M4 are large IB4^-^ neurons (C_m_ is >60pF), have a pronounced “spike-like” peak on their outward current, similar to M1 and M3 (Fig 4G), and do not respond to ATP, CAP, MO or 5-HT (Fig 3). The distinct feature of M4 neurons is a very fast dB of AP, but relatively slow AHP (Fig 3). M4 also differed from M1/M1a and M3/M3a in the falling phase of AP (Fig 4D). M4 has a very fast falling phase with no deflection; and FT:RT ratio is always <1 for M4, and >1 for M1/M1a and M3/M3a (Table 1). Analysis of properties of marker expressing neurons has unexpectedly shown that M4 is one of two prominent groups expressing trkC^cre-ER^/TdTomato (Table 5).

### TRPV1^cre^/TdTomato and TRPV1-GFP expressing sensory neuronal groups

Recording and analysis data from 88 DRG neurons of TRPV1^cre^/TdTomato reporter mice showed that many TRPV1-cre^+^ neuronal groups (highlighted with blue in Table 3) were not responsive to CAP. This phenomenon could be due to fate map and has been previously reported [19,49]. Moreover, TRPV1-cre^+^ is present in all peptidergic CGRP^+^ DRG neuronal groups (i.e. small and medium-large neurons), as well as non-peptidergic nociceptors (Table 3). Overall, TRPV1^cre^/TdTomato slightly differs from the Nav1.8^cre^/TdTomato reporter expression pattern in DRG neurons, since as TRPV1-cre^+^ cells were not detected in S7 or M4 neuronal groups (Table 3).

Fifty nine TRPV1-GFP (V1-GFP^+^) DRG neurons stained with IB4 were recorded with sequential protocols as described in “Methods”. All but 2 V1-GFP^+^ neurons could be assigned to one of 4 main clusters, each of which belonged to small-sized neuronal groups (Table 4). Every V1-GFP^+^ neuron is responsive to CAP, and the strongest I_CAP_ was found in S6 CGRP-cre^-^ neurons. Generally, all V1-GFP^+^ neuronal groups apart from S6 are peptidergic neurons and represented in CGRP-cre^+^ DRG neurons from CGRP^cre-ER^/TdTomato mice. Moreover, all V1-GFP^+^ neuronal groups are denoted in Nav1.8-cre^+^ and TRPV1-cre^+^ DRG neurons from Nav1.8^cre^/TdTomato and TRPV1^cre^/TdTomato mice, respectively.

### Sensory neuronal groups not expressing Nav1.8-cre

Nav1.8-cre^+^ includes all of the CGRP-cre^+^ and TRPV1-cre^+^ neuronal groups. Hence, only 45 Nav1.8-cre^-^ DRG neurons were selected for characterization of electrophysiological profiles. Fifteen of 45 Nav1.8-cre^-^ neurons fit into neuronal group M2 (Table 1). M2 is IB4^-^ and non-responsive to ATP, CAP, MO and 5-HT. M2 has fast AP with FT:RT<1. ≈70% of M2 neurons exhibited an uncommon AHP shape that did not show the classical “overshoot” below V_m_ (Fig 5). The remaining M2 neurons had a very small overshoot and a fast AHP (Fig 5). All M2 neurons also displayed a biphasic I with a combination of small “spike-like” peak and a distinctive “smooth I curve”, which was typical for S1-S5 neurons (Figs 4F and 5). Analyses of electrophysiological profiles of trkB-cre^+^ neurons from trkB^cre-ER^/TdTomato mice implied that M2 matches a profile of trkB-cre^+^ neurons (Table 5).

Ten of 45 Nav1.8-cre^-^ neurons fit into neuronal group M5 (Table 1). Like M2, M5 is IB4^-^ and non-responsive to CAP or MO. However, some of M5 neurons show small (<0.3 nA) I_5HT_. M5 had a fast AP with FT:RT<1 and the AHP quickly returned to V_m_ level (Fig 5). The main distinctions of M5 from M2 are that M5 has medium-sized fast I_ATP_; and “spike-like” peak from recording of I (Fig 5). TrkC-cre^+^ neurons isolated from DRG of trkC^cre-ER^/TdTomato mice could be divided into two main and distinct groups. One of these trkC-cre^+^ neuronal groups has matched M5, while another trkC-cre^+^ group is M4 (Table 5).

Four of 45 Nav1.8-cre^-^ neurons were assigned to the M6 group, AP and AHP of which bears strong similarity to some of M2 neurons. M6 group neurons have fast AHP with shallow “overshoot” of AP below V_m_ (Fig 5). However, the distinction between M2 and M6 is that the M6 outward current does not have a “spike-like” peak. Moreover M6 neurons exhibited I_ATP_, similar to those produced in M5 neurons (Fig 5). We could not identify the apparent marker for M6 neurons.

Sixteen of 45 Nav1.8-cre^-^ neurons fit into neuronal group M7 (Table 1). M7 is IB4^-^ and non-responsive to ATP, CAP, MO or 5-HT. M7 have the fastest AHP and AP with FT:RT<1 compared to the other sensory neuron groups (Fig 5). Moreover, the “overshoot” below V_m_ was largest for medium-large sensory neurons (Fig 5). M7 I has the characteristic “spike-like” peak, but it does not fit well with the exponential equation and yields high τ values (Fig 5). Analysis of parvalbumin (PV-cre^+^) neurons from PV^cre^/TdTomato^+^ mice showed that there are two PV-cre^+^ groups. One of them is similar to M7 neuronal group (Table 5). Another PV-cre^+^ group has a profile matching M5 groups (see above). This suggests that M5 neurons are PV^+^/trkC^+^.

### Immunohistochemical (IHC) analysis of CGRP-cre^+^ DRG neurons

**Fig 6:**
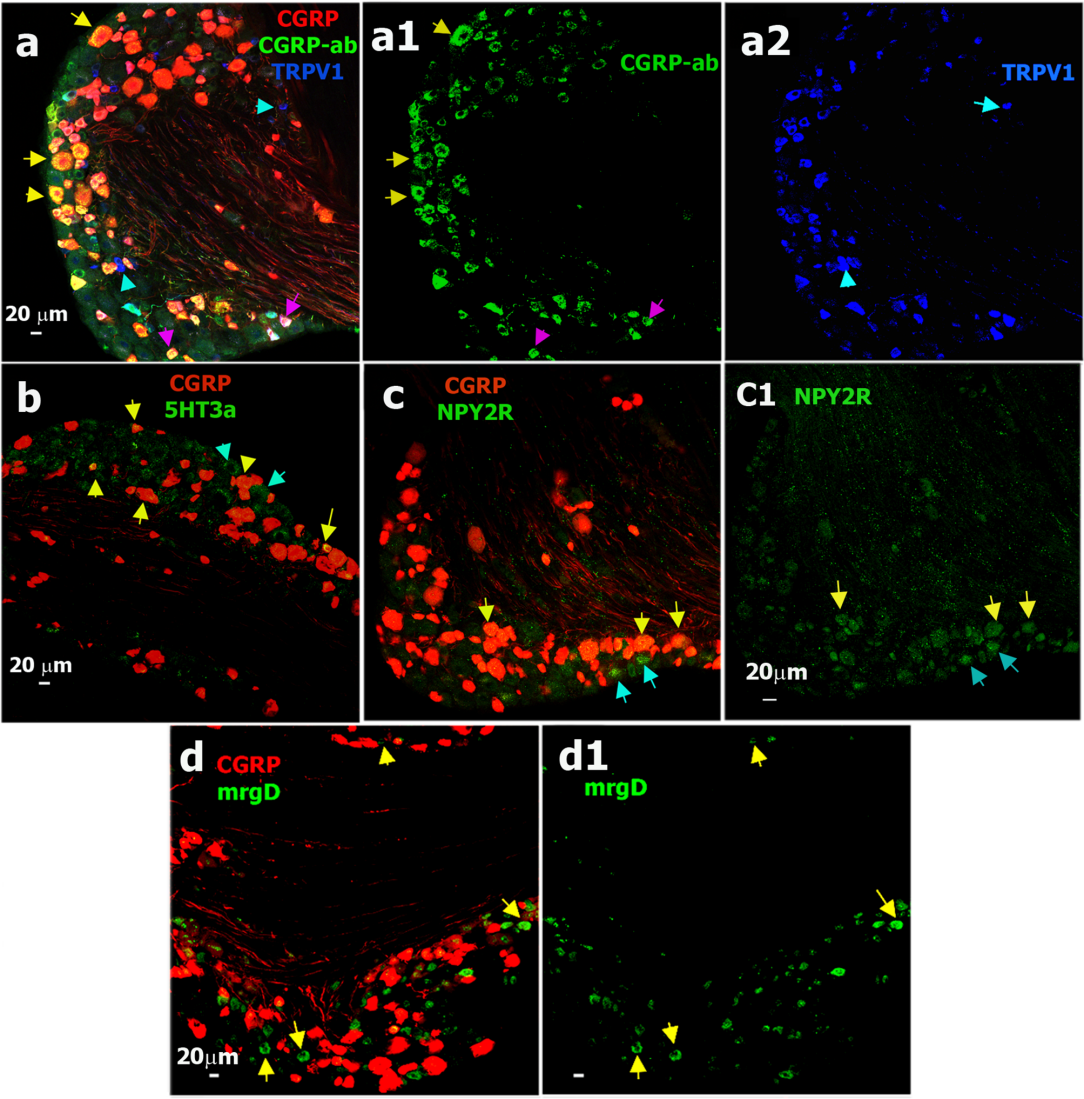
Sensory neuronal markers expressing in CGRP-cre^+^ DRG neurons. **a.** Expression of CGRP-ab (green) and TRPV1 (blue) relatively CGRP-cre^+^ neurons (red) in DRG from CGRP^cre-ER/+^;Rosa26^LSL-tDTomato/+^ mice. Medium-large CGRP-cre^+^/CGRP-ab^+^ neurons are marked with yellow arrows (**a** and **a1**); small CGRP-cre^+^/CGRP-ab^+^ neurons are marked with purple arrows (**a** and **a1**); and small CGRP^-^/TRPV1^+^ neurons (<20μm) are marked with sapphire arrows (**a** and **a2**). **b.** Expression of 5HT3a (green) relative to CGRP-cre^+^ neurons (red) in DRG. CGRP-cre^+^/5HT3a^+^ neurons are marked with yellow arrows and CGRP-cre^-^/5HT3a^+^ neurons are marked with sapphire arrows. **c.** Expression of NPY2R (green) relative to CGRP-cre^+^ neurons (red) in DRG. Medium NPY2R^+^/CGRP-cre^+^ neurons are marked with yellow arrows (**c** and **c1**) and medium NPY2R^+^/CGRP-cre^-^ neurons are marked with sapphire arrows (**c** and **c1**). **d.** Expression of mrgD (green) relative to CGRP-cre^+^ DRG neurons (red). Yellow arrows mark CGRP-cre^-^/mrgD^+^ neurons (**d** and **d1**). White horizontal bar shows 20μm scale for each panel.

To further characterize the CGRP^cre-ER/+^;Rosa26^LSL-tDTomato/+^ transgenic line and confirm our electrophysiology data, we used IHC to examine expression of sensory neuronal markers in L3-L5 lumbar DRG sections from this mouse line. CGRP antibodies (CGRP-ab^+^) labeled 48.2±3.1%, and CGRP-cre^+^ (i.e. red neurons from CGRP^cre-ER^/TdTonato mice) were counted in 44.5±2.6% of all DRG neurons (Figs 6A, 6a1, 7 and 8B). It demonstrate that systemically applied tamoxifen induced effective *cre*-recombination, generating CGRP^+^ neurons in 84.5±4.9% of CGRP-ab^+^ DRG neurons (Figs 6A, 6a1 and 8B). Consistent with our electrophysiology data, 58% CGRP-cre^+^ neurons had TRPV1 immunoreactivity (Fig 6A and 6a2), and 26% and 10% of CGRP-cre^+^ cells co-express 5HT3a and NPY2R, respectively (Figs 6B, 6C, 6c1 and 8C). However, 25.9±6.2% of TRPV1^+^ neurons were CGRP-cre^-^ (Fig 6A and 6a2). About 30% of weakly 5HT3a^+^ neurons were CGRP-cre^-^ (Fig 6B); and approximately 30% of NPY2R^+^ neurons did not have CGRP *cre*-recombination (Fig 6C and 6c1). This finding is in agreement with a report where 25% NPY2R^+^ cells did not express CGRP [5].

**Fig 7:**
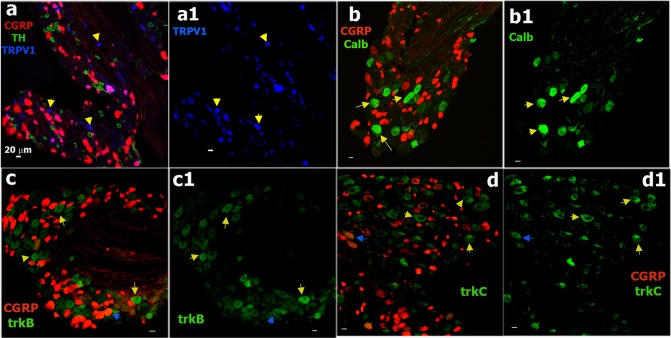
Sensory neuronal markers non-expressing in CGRP-cre^+^ DRG neurons. **a.** Expression of TH (green) and TRPV1 (blue) relatively to CGRP-cre^+^ neurons (red) in DRG from CGRP^cre-ER/+^;Rosa26^LSL-tDTomato/+^ mice. Some TRPV1^+^ DRG neurons are not expressed in CGRP-cre^+^ sensory neurons. These CGRP-cre^-^/TRPV1^+^ neurons are marked with yellow arrows (**a** and **a1**). **b.** Expression of calbindin-28K (Calb; green) relatively to CGRP-cre^+^ DRG neurons (red). Yellow arrows mark CGRP-cre^-^/Calb^+^ neurons (**b** and **b1**). **c.** Expression of trkB (green) relatively to CGRP-cre^+^ DRG neurons (red). Yellow arrows mark CGRP-cre^-^/trkB^+^ neurons; and a blue arrow shows a rare example of the CGRP-cre^+^/trkB^+^ neuron (**c** and **c1**). **d.** Expression of trkC (green) relatively to CGRP-cre^+^ DRG neurons (red). Yellow arrows mark CGRP-cre^-^/trkC^+^ neurons; and; and a blue arrow shows an example of the CGRP-cre^+^/trkC^+^ neuron (**d** and **d1**). White horizontal bar shows 20μm scale for each panel.

**Fig 8:**
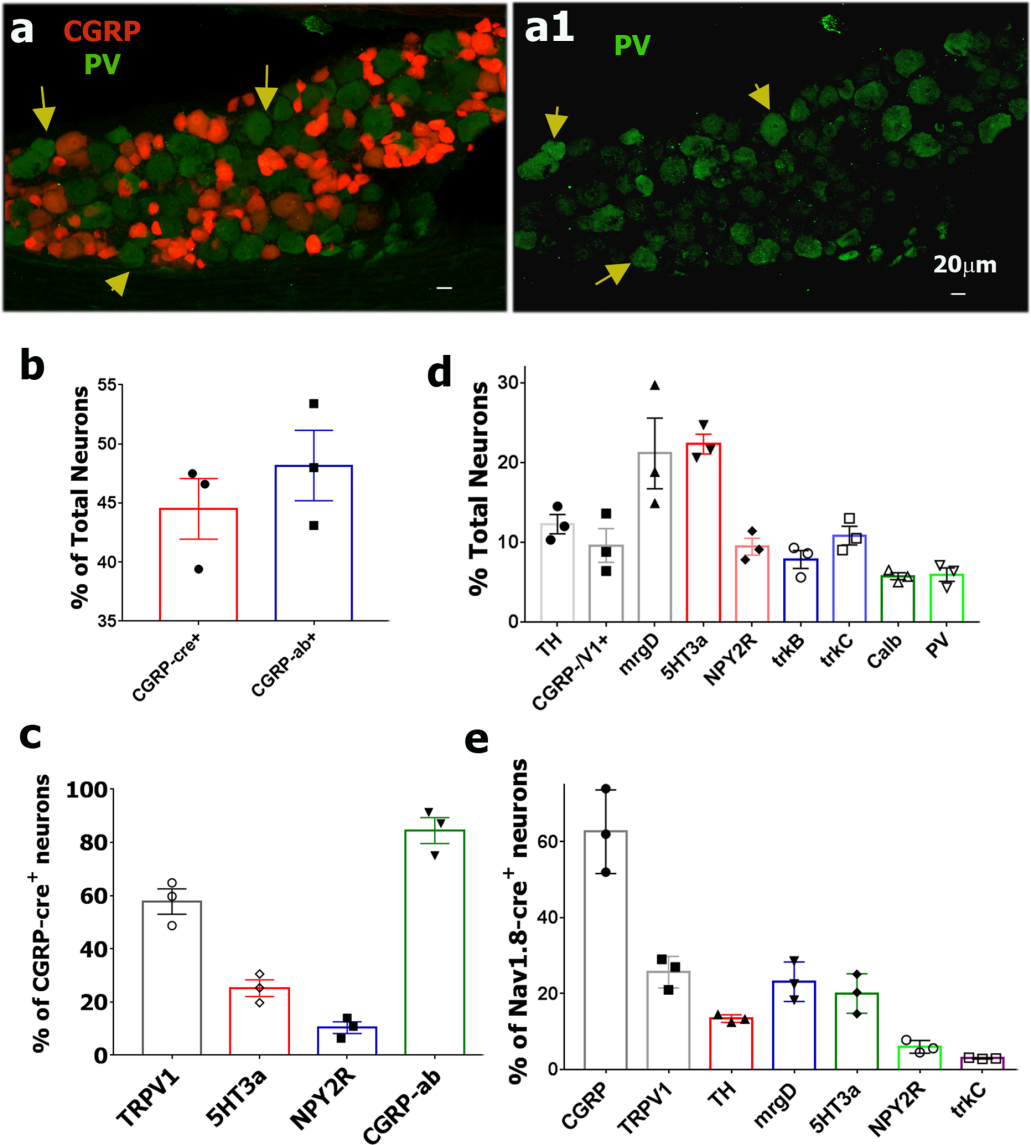
Expression pattern of sensory neuronal markers in CGRP-cre^+^ and Nav1.8-cre^+^ DRG neurons. **a.** Expression of parvalbumin (PV; green) relative to CGRP-cre^+^ DRG neurons (red). Yellow arrows mark CGRP-cre^-^/PV^+^ neurons (**a** and **a1**). White horizontal bar shows 20μm scale for each panel. **b.** Percentages of total L3-L5 DRG neurons labeled in CGRP^cre-ER/+^; Rosa26^LSL-tDTomato/+^ mice (i.e. CGRP-cre^+^), and with CGRP antibodies (i.e. CGRP-ab^+^). Cell counting is from three animals, 3–4 sections each. **c.** Percentage of CGRP-cre^+^ L3-L5 DRG neurons in CGRP^cre-ER/+^;Rosa26^LSL-tDTomato/+^ mice labeled with indicated neuronal markers. Cell counting is from three animals, 4–5 sections each. **d.** Percentages of total L3-L5 DRG neurons labeled with a variety of indicated sensory neuronal markers. Cell counting is from three animals, 3–5 sections each. **e.** Expression percentages of markers in Nav1.8-cre^+^ in L3-L5 mouse DRG neurons. Cell counting is from three animals, 3–5 sections each.

Electrophysiology showed several neuronal groups that did not display CGRP^+^ cre-recombination. We performed IHC on L3-L5 DRG from CGRP^cre-ER/+^;Rosa26^LSL-tDTomato/+^ with markers for these groups (Fig 7). 10% of TRPV1^+^ neurons, most likely representing the S6 group, were CGRP-cre^-^ (Figs 6A, 6a2 and 8D; Tables 1 and 5). 21% of mrgD^+^ cells (S4 and S5 groups), which are non-peptidergic C-fiber nociceptors [7], did not express in CGRP-cre^+^ neurons (Figs 6D, 6d1 and 8D). CGRP-cre^+^ was almost absent in 12% of TH^+^ (Figs 7A, 7a1 and 8D); 8% of trkB^+^ (Figs 7C, 7c1 and 8D); 11% of trkC^+^ (Figs 7D, 7d1 and 8D); 6% of calbindin (Calb^+^) (Figs 7B, 7b1 and 8D) and 6% of PV^+^ neurons (Figs 8A, 8a1 and 8D). Overall, IHC closely matches electrophysiology data in regards to the occurrence of CGRP^+^ *cre*-recombination in a subset of TRPV1^+^ (groups S1, S2 and S3), 5HT3a^+^ (S3 and M1/M1a groups) and NPY2R^+^ (M3/M3a group) neurons. Finally, CGRP-cre^+^ was hardly detected in a subset of TRPV1^+^ (S6 group), mrgD^+^ (S4 and S5 groups), trkB^+^ (M2 group), trkC^+^ (M4 and M5 groups), Calb^+^ (probably M6 group) and PV^+^ (M5 and M7 groups) DRG neurons.

### IHC analysis of Nav1.8-cre^+^ DRG neurons

**Fig 9:**
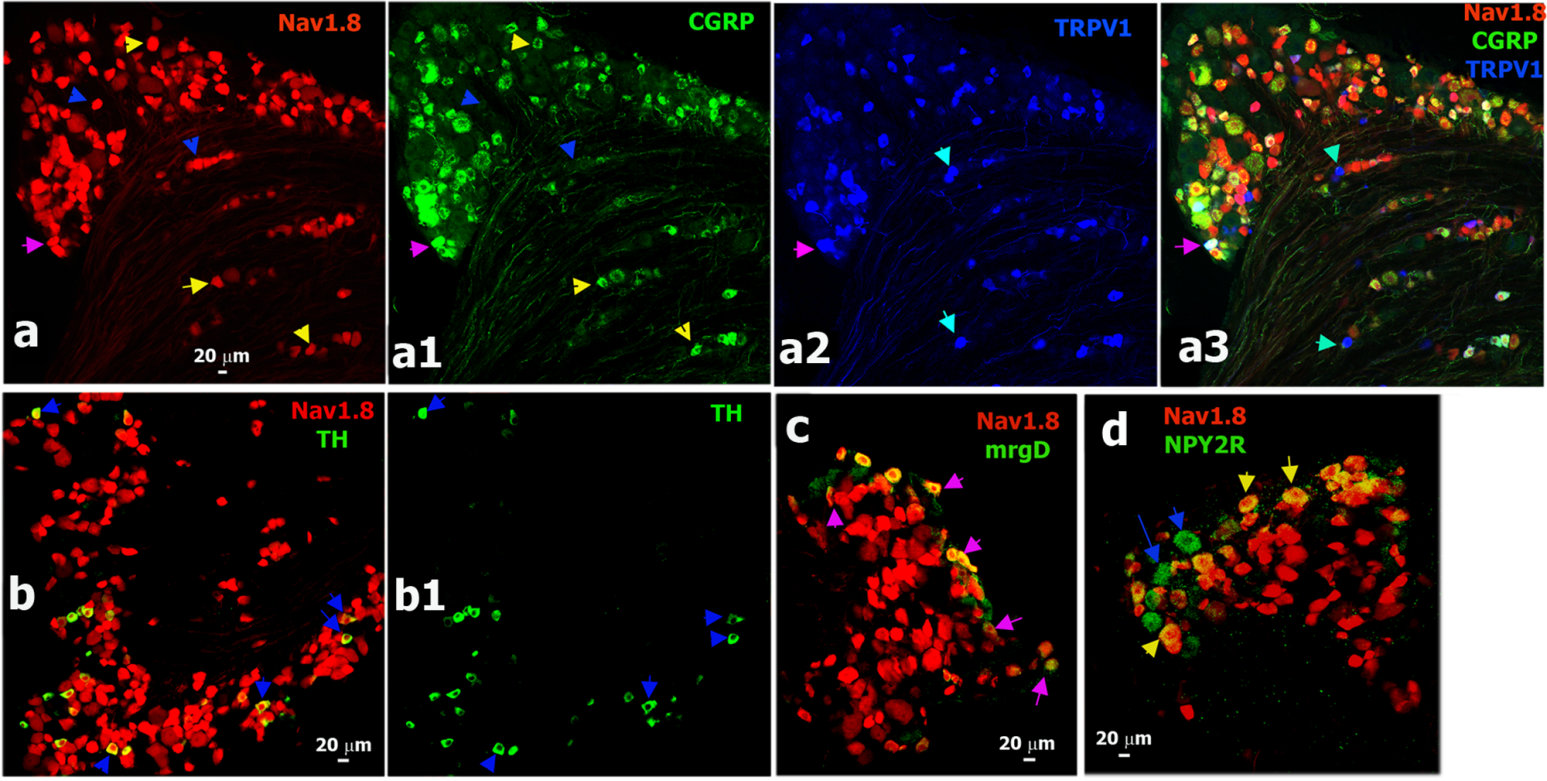
Sensory neuronal markers expressing in Nav1.8-cre^+^ DRG neurons. **a.** Expression of CGRP (green) and TRPV1 (blue) in Nav1.8-cre^+^ DRG neurons (red) in L3-L5 DRG of Nav1.8^cre/+^;Rosa26^LSL-tDTomato/+^ mice. Sapphire arrows mark TRPV1^+^/Nav1.8-cre^-^ neurons (**a2** and **a3**); a pink arrow marks a TRPV1^+^/CGRP^+^/Nav1.8-cre^+^ neuron (**a-a3**), yellow arrows mark CGRP^+^/Nav1.8-cre^+^ neurons (**a** and **a1**) and blue arrows mark CGRP^-^/Nav1.8-cre^+^ neurons (**a** and **a1**). **b.** Co-expression of TH (green) and Nav1.8-cre^+^ (red) neurons in DRG. Blue arrows mark TH^+^/Nav1.8-cre^+^ neurons (**b** and **b1**). **c.** Co-expression of MrgprD (green) and Nav1.8-cre^+^ (red) neurons in DRG. Pink arrows mark mrgD^+^/Nav1.8-cre^+^ neurons. **d.** Co-expression of NPY2R (green) and Nav1.8-cre^+^ (red) neurons in DRG. Blue arrows mark NPY2R^+^/Nav1.8-cre^-^ neurons and yellow arrows mark NPY2R^+^/Nav1.8-cre^+^ neurons. White horizontal bar shows 20μm scale for each panel.

**Fig 10:**
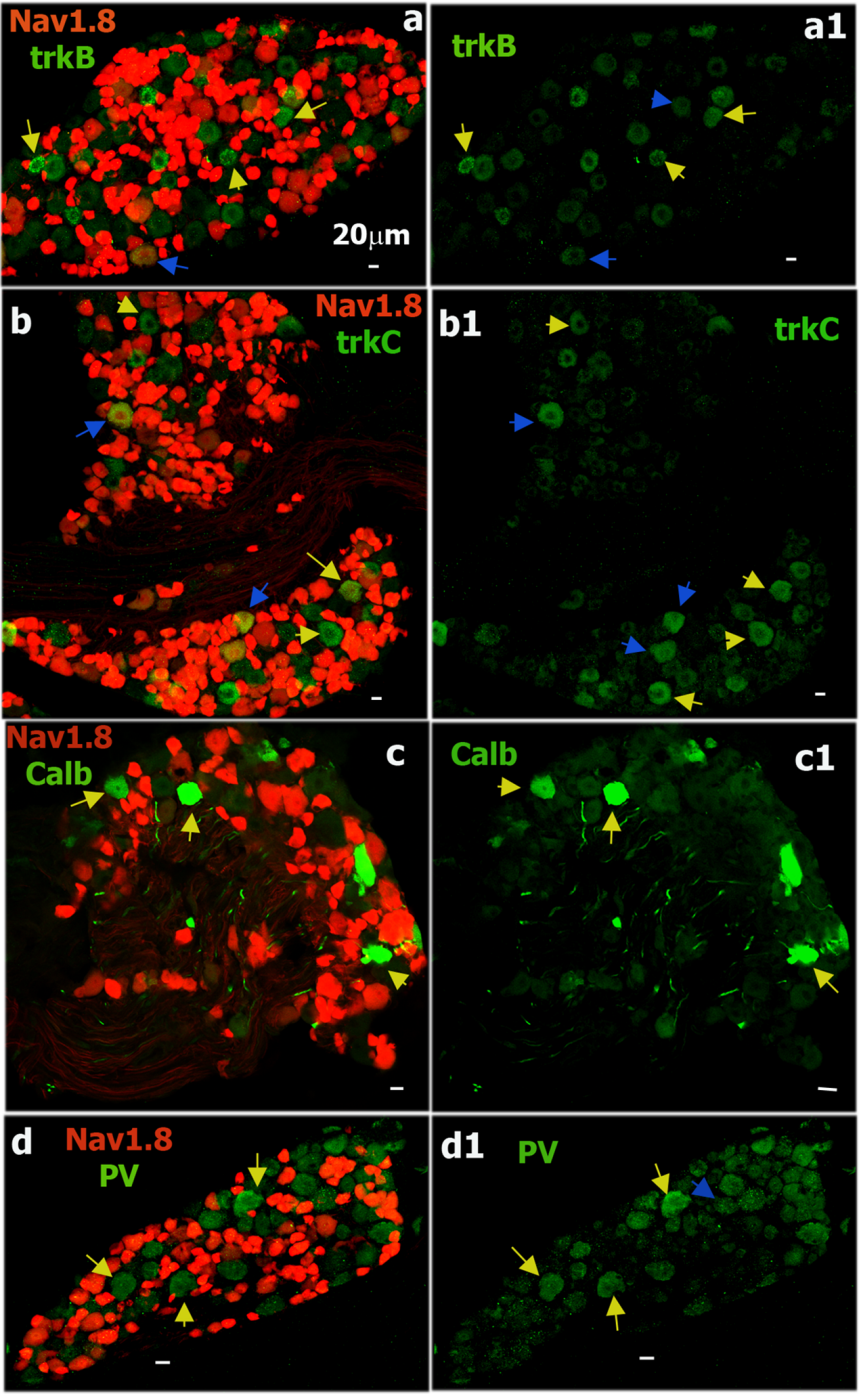
Sensory neuronal markers non-expressing in Nav1.8-cre^+^ DRG neurons. **a.** Expression of trkB (green) and Nav1.8-cre^+^ (red) neurons in Nav1.8^cre/+^;Rosa26^LSL-tDTomato/+^ mouse L3-L5 DRG. Yellow arrows mark trkB^+^/Nav1.8-cre^-^ neurons, and blue arrows show few trkB^+^/Nav1.8-cre^+^ neurons (**a** and **a1**). **b.** Expression of trkC (green) and Nav1.8-cre^+^ (red) neurons in Nav1.8^cre/+^;Rosa26^LSL-tDTomato/+^ mouse L3-L5 DRG. Yellow arrows mark trkC^+^/Nav1.8-cre^-^ neurons and blue arrows mark trkC^+^/Nav1.8-cre^+^ neurons (**b** and **b1**). **c.** Expression of calbindin (Calb; green) and Nav1.8-cre^+^ (red) neurons in Nav1.8^cre/+^;Rosa26^LSL-tDTomato/+^ mouse L3-L5 DRG. Yellow arrows mark Calb^+^/Nav1.8-cre^-^ neurons (**c** and **c1**). **d.** Expression of parvalbumin (PV; green) and Nav1.8-cre^+^ (red) neurons in Nav1.8^cre/+^;Rosa26^LSL-tDTomato/+^ mouse L3-L5 DRG. Yellow arrows mark PV^+^/Nav1.8-cre^-^ neurons and a blue arrow marks a rare example of PV^+^/Nav1.8-cre^+^ neurons. White horizontal bar shows 20μm scale for each panel.

We next performed IHC with antibodies against a number of neuronal markers on L3-L5 DRG sections from the Nav1.8^cre/+^;Rosa26^LSL-tDTomato/+^ transgenic line to examine the expression pattern of Nav1.8-cre^+^ neurons (Figs 9 and 10). Nav1.8-cre^+^ neurons were 82.7±2.6% of all neurons in DRG (Fig 9A). CGRP antibodies labeled 62.7±6.4% of Nav1.8-cre^+^ neurons (Figs 9a1, 9a3 and 8E). None of the CGRP-ab^+^ neurons was found outside of the Nav1.8-cre^+^ subset. TRPV1 was present in 26% of Nav1.8-cre^+^ DRG neurons (Figs 9A, 9a2, 9a3 and 8E). Unlike CGRP antibodies, surprisingly, a small portion of neurons labeled with TRPV1 antibodies (4.7±0.7% of all DRG neurons) was not co-expressed with Nav1.8-cre^+^ (Fig 9a2 and 9a3). Interestingly, all of TRPV1^+^/Nav1.8-cre^-^ neurons were also negative to CGRP antibodies (Fig 9A–9a3). Since TRPV1^+^/CGRP^-^ neurons (i.e. S6 group) are ≈11% of all neurons and TRPV1^+^/CGRP^-^/Nav1.8-cre^-^ neurons are ≈4.5% of all neurons, then this indicates that ≈40% of S6 group neurons could be Nav1.8-cre^-^ (Tables 1–3). In concordance with electrophysiology data (Table 3), 13% Nav1.8^+^ neurons were co-labeled with anti-TH (Figs 9B, 9b1 and 8E). A majority (>90%) of non-peptidergic nociceptors, MrgprD^+^, and 5HT3a^+^ nociceptors were also within a Nav1.8-cre^+^ subset (Fig 9C and 9D). MrgprD^+^ and 5HT3a^+^ neurons composed 23.1±3.0% and 20.0±3.1% of a Nav1.8-cre^+^ subset, respectively (Fig 8E). NPY2R^+^ neurons account for 6% of the Nav1.8-cre^+^ subset (Figs 8E and 9D). Interestingly, a bulk of NPY2R^+^/Nav1.8^+^ neurons had weaker red labeling. Weak red Nav1.8^+^ labeling was also observed in a subset of trkC^+^ neurons (Figs 9D, 10B and 10b1). There was small percentage of trkC^+^ among the Nav1.8-cre^+^ neurons (2.9±1.1%; Figs 8E, 10B and 10b1). Since some of Nav1.8-cre^+^ neurons exhibit fast AP with very slow AHP (Table 3), it could be that Nav1.8-cre^+^/trkC^+^ neurons belong to M4 group (Table 5). Finally, IHC confirmed electrophysiology data (Tables 1 and 3) showing that several sensory neuronal groups, such as trkB^+^ (Fig 10A and 10a1), ≈70% of trkC^+^ (Fig 10B and 10b1), Calb^+^ (Fig 10C and 10c1) and PV^+^ (Fig 10D and 10d1) hardly co-express with Nav1.8-cre^+^ neurons. In summary, IHC, in accordance with electrophysiology, demonstrated that Nav1.8^cre/+^ is able to make *cre*-recombination in almost all small-sized neurons (i.e. peptidergic and non-peptidergic; S1-S6 groups), subset of medium-sized neurons (M1/M1a and M3/M3a groups), tiny TH^+^ neurons (S7 group) and a subset of large trkC^+^ neurons (M4 group). Finally, Nav1.8-cre^+^ neurons were seldom found among trkB^+^ (M2 group), trkC^+^ (M5 group), Calb^+^ (probably M6 group) and PV^+^ (M5 and M7 groups) neurons.

## Discussion

Nav1.8^cre/+^ and TRPV1^cre/+^ reporter mice have been extensively used to manipulate subsets of sensory neurons and/or ablate a variety of molecules/targets in these subsets [18,19]. Nav1.8^cre/+^ was generated with intention to target C-nociceptive neuronal groups, since original studies supported predominant localization of Nav1.8 to small-medium diameter neurons [44]. Following studies showed that Nav1.8 is expressed in neurons with diameters ranging from 20 to 70 μm [50,51]. Investigation of subsets of Nav1.8-cre^+^ neurons identified in Nav1.8^cre/+^/TdTomato mice showed in ≈82% of all DRG neurons, and almost all CGRP^+^ neurons are Nav1.8-cre^+^ [52]. However, precise identities for medium and large-sized Nav1.8-cre^+^ sensory neurons were not detailed. To answer these questions, we used IHC with sensory neuronal markers and well described patch-clamp electrophysiology approaches [21,45] to characterize DRG neuronal groups expressing GFP or TdTomato under control of CGRP^cre-ER/+^, Nav1.8^cre/+^, TRPV1^cre/+^ and TRPV1-GFP reporters. We then identified these neuronal groups by matching their properties to electrophysiological profiles of DRG neurons from reporter mice expressing well-established sensory neuronal markers.

TRPV1^cre/+^ was produced for the manipulation of CAP responsive sensory neurons. However, this and previous studies showed that TRPV1-cre^+^ DRG neurons identified TRPV1^cre/+^/TdTomato mice are often CAP non-responsive. This could occur due to a fate map of TRPV1 expression in DRG sensory neurons during embryogenesis. Therefore, we also employed TRPV1-GFP mouse line as a reference point, since GFP expression is under control of the TRPV1 promoter, and is not affected by alterations in TRPV1 expression during development. In addition, presented detailed study of TRPV1-cre^+^ neuronal groups in TRPV1^cre/+^/TdTomato mice revealed that TRPV1^cre/+^ line could be optimal in manipulation of all nociceptive neurons disregarding of their sizes.

Peptidergic neurons were previously studied using CGRP-GFP [53] and recently generated CGRP-mCherry reporter mouse lines [54]. Functional study is showed that some of CGRP^+^ neurons are insensitive to mechanical nociceptive stimuli [54]. It was also shown that CGRP-GFP^+^ neurons in CGRP-GFP mice respond to CAP, MO, menthol, acidic pH and ATP. 50% of CGRP-GFP^+^ neurons from CGRP-GFP mice are expressed TRPV1, 25% have been labeled with neurofilament-200 (NFH), and 10% contained IB4 and almost none overlapped with TRPM8 [53]. These data is congruent with results presented in this study. However, certain questions (such as what CGRP-GFP^+^ neuronal groups are NFH^+^; how many CGRP-GFP^+^ groups are TRPV1^+^, and do they express TRPA1^+^) are still remained. Besides, CGRP-GFP and CGRP-mCherry reporter mouse lines are not optimal for generation conditional knock-outs, and ablation of CGRP^+^ subsets and manipulation of CGRP^+^ subsets with ontogenetic approaches. Presented study in detailed characterized CGRP-cre^+^ neurons using alternative CGRP^cre/+-ER^ inducible mouse reporter line, which can be used for genetic ablation of specific genes in CGRP^+^ subsets and manipulation of CGRP^+^ neurons and afferents.

**Fig 11:**
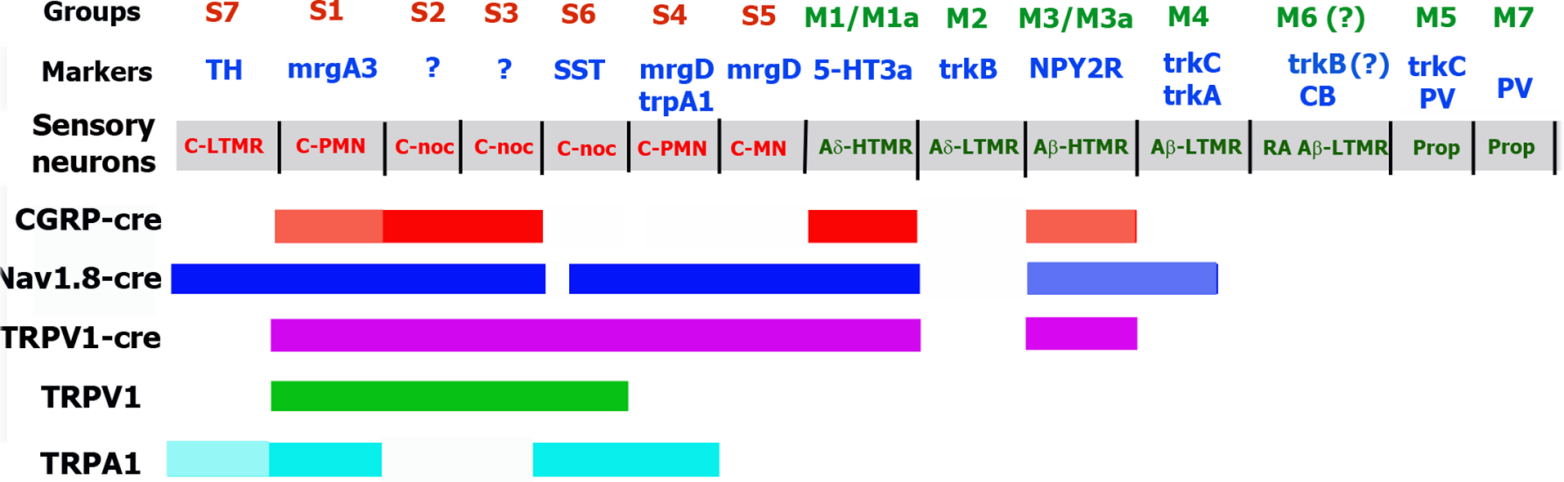
Schematic representation of CGRP-cre^+^, Nav1.8-cre^+^, TRPV1-cre^+^ and V1-GFP^+^ subsets. Schematic representation of expression pattern for CGRP-cre^+^ in L3-L5 DRG of CGRP^cre-ER/+^;Rosa26^LSL-tDTomato/+^ mice; for Nav1.8-cre^+^ in L3-L5 DRG of Nav1.8^cre/+^;Rosa26^LSL-tDTomato/+^; for TRPV1-cre^+^ in L3-L5 DRG of TRPV1^cre/+^;Rosa26^LSL-tDTomato/+^ and for V1-GFP^+^ in L3-L5 DRG of TRPV1-GFP reporter mice. MO responsive subsets are indicated as TRPA1-postive. “Groups” line represents name of sensory neuronal groups presented in this study (see Table 1). “Marker” line signifies distinctive markers for each group. If marker is not defined for particular sensory neuronal group, “?” sign was used. “Sensory neurons” indicates possible functions of sensory neuronal groups in the grey box. C-PMN is C-polymodal nociceptor; C-noc is C-nociceptors (modalities are unknown); C-MN is C- mechano-nociceptors; C-LTMR is C-low threshold mechano-receptor; Aδ-HTMR is Aδ-high threshold mechano-receptor; Aδ-LTMR is Aδ-low threshold mechano-receptor; Aβ-HTMR is Aβ-high threshold mechano-receptor; Aβ-LTMR is Aβ-low threshold mechano-receptor; RA Aβ-LTMR is rapid adapting Aβ-low threshold mechano-receptor; and Prop is proprioceptors.

Using reporter mice driven by promoters of well-characterized sensory neuronal markers, CGRP-cre^+^, Nav1.8-cre^+^, TRPV1-cre^+^ or V1-GFP^+^ sensory neuronal groups could be assigned to particular cluster identified by single-cell transcriptomic, or allotted to functional group investigated with use of a particular marker-reporter mouse line. Thus, S1 group expressing CGRP-cre^+^, Nav1.8-cre^+^, TRPV1-cre^+^ and V1-GFP^+^ matches MrgA3-cre^+^ neurons, which were established as C-polymodal nociceptors (C-PMN; Fig 11) [55,56]. Based on single-cell sequencing of mRNA from DRG neurons, S1 likely corresponds to NP2 (Table 5) [1]. Precise function of S2 and S3, which express CGRP-cre^+^, Nav1.8-cre^+^, TRPV1-cre^+^ and V1-GFP^+^, are unknown. However, they could likely be assigned together to PEP-1 group (Fig 11; Table 5) [1]. S4 and S5 groups, which have both Nav1.8-cre^+^ and TRPV1-cre^+^, but not CGRP-cre^+^, are classic CAP-unresponsive IB4^+^ non-peptidergic sensory neurons [48]. These neurons are identified as MrgprD^+^ [7,57]. Functional studies distinguished two types of MrgprD^+^ neurons: C-PMN and C-mechano-nociceptors (C-MN) [57,58]. Electrophysiological profiles distinguish two MrgprD^+^ groups: S4 and S5, but it is not clear which group is C-MN (Fig 11). Single-cell sequencing does not distinguish between MrgprD^+^ groups (i.e. S4 and S5), marking them as the NP1 group (Table 5) [1]. S6 group is non-peptidergic, express TRPV1, and some S6 neurons (≈40%) lack Nav1.8-cre. We did not find clear markers for the S6 neuronal group. However, very small-sized TRPV1^+^/CGRP-cre^-^/IB4^-^, and CAP and MO responsive S6 neurons could resemble to somatostatin-positive neurons (NP-3 group) revealed by single-cell transcriptomics (Fig 11; Table 5) [1]. Function of S6/NP-3 neurons is unknown. Non-peptidergic and Nav1.8-cre^+^ S7 neurons represent vGLUT3-cre^+^ groups, which has been defined as C-low threshold mechano-receptors (C-LTMR; Fig 11) [6]. S7 neurons have another marker TH and are assigned to TH-group by single-cell transcriptomics (Table 5) [1,10]. We have found that some of vGLUT3-cre^+^ neurons have C_m_ of >50pF. Medium-sized vGLUT3-cre^+^ neurons were also reported in original publication [6]. In contrast, TH antibodies consistently label very small-to-small sized neurons (Fig 9B and 9b1) [1,10]. This would therefore imply that most TH^+^ neurons are identical to vGLUT3-cre^+^ neurons, and transcriptomic data point out that TH is predominantly expressed in C-LTMR [1]. Medium-sized peptidergic neurons are represented by M1/M1a and M3/M3a groups. Base on marker expression and single-cell sequencing, M1/M1a and M3/M3a neurons could be peptidergic myelinated A-fiber nociceptors [1]. Properties and marker expression imply that NPY2R^-^/5HT3a^+^ M1/M1a neurons could likely be Aδ-high threshold mechano-receptors (HTMR), and M3/M3a, which are NPY2R^+^, could probably represent separate group of A-fiber HTMR, which are sometime designated as Aβ-HTMR (Fig 11) [5,22]. Single-cell based sequencing did not distinguish these groups, and both of them were included in PEP-2 (Table 5) [1]. M2 neurons do not contain CGRP-cre, Nav1.8-cre or TRPV1-cre; and are matched to trkB-cre^+^ neurons, which could be assigned to NF1 group (Table 5) [1]. Functional analysis showed that trkB-cre^+^ neurons belong to Aδ-LTMR family (Fig 11) [9]. M4 group is matched to a group expressing trkC-cre, and has some similar features to NF-3 neurons (Table 5) [1]. Interestingly, M4 neurons have Nav1.8-cre. However, the function of M4 neurons is not apparent. Besides M2 group, there are several other neuronal groups that is not revealed in CGRP^cre-ER/+^, Nav1.8^cre/+^, TRPV1^cre/+^ or TRPV1-GFP reporter mice. Thus, M5 neurons are trkC-cre^+^/PV-cre^+^. Functional studies suggested that trkC^+^/PV^+^ are either Aβ-LTMRs or proprioceptors (Fig 11) [46]. Single-cell transcriptomics allotted this group as NF4 or NF5 group (Table 5) [1]. Electrophysiological profile showed that the M7 group are likely classical proprioceptors trkC-cre^-^/PV-cre^+^ [22] and can be classified as either in the NF4 or NF5 group (Table 5) [1]. We have not analyzed DRG neurons from calbindin (Calb) reporter mouse. Single-cell transcriptomic assigned Calb^+^ neurons to group NF2, which also expresses trkB at a lower level [1]. Functional studies on Calb^+^ neurons demonstrated that they are Aβ-LTMR neurons [5]. It could be that M6 group parallels to NF2 and belong to rapid adapting Aβ-LTMR neurons (Fig 11) [1,5].

Overall, the presented data indicate that tamoxifen-induced *cre*-recombination in the CGRP^cre-ER/+^ reporter mice occurs in 84% of peptidergic (i.e. CGRP-ab^+^) neurons. Possible leak into non-peptidergic nociceptors or LTMR-classified neurons is minimal if any (Table 2; Fig 11). Our electrophysiology and IHC data is consistent with results of single-cell sequencing, leading to the conclusion that CGRP-cre^+^ neurons (S1-S3, M1/M1a and M3/M3a groups) are likely belonging to PEP-1, PEP-2 and NP-2 clusters (Table 5) [1]. Nav1.8^cre/+^-driven *cre*-recombination happens in 82% of all DRG neurons, which encompasses all peptidergic C- and A-nociceptors, all non-peptidergic C-nociceptors (S4-S6 groups) as well as some LTMRs (S7 and M4 groups) (Table 3; Fig 11) [6]. Match to single-cell sequencing data revealed that Nav1.8-cre^+^ neurons probable belong to PEP-1, PEP-2, NP-1, NP-2, NP-3 and NF-3 groups (Table 5) [1]. Neurons labeled in TRPV1^cre/+^/TdTomato reporter mice include all peptidergic and non-peptidergic C- and A-nociceptors, but no LTMR neurons. This makes TRPV1^cre/+^ an optimal reporter mouse line for manipulation of all nociceptors (Table 4; Fig 11).

Despite dominat expression of CGRP and TRPV1 in sensory neurons, and many advantages of CGRP^cre-ER/+^ and TRPV1^cre/+^ reporter mice in the manipulation of sensory neurons, TRPV1 and to lesser extent CGRP expression could be detected outside sensory neurons. Thus, TRPV1 is expressed at low-to-medium levels in the immune system [59], epithelial cells [60] and other neurons of the CNS [61]. Outside sensory neurons in naïve animals, CGRP medium-to-high levels of expression are localized to specific subsets of neuronal and non-neuronal cells [53,62]. Thus, pulmonary neuroendocrine epithelial cells express CGRP on very high levels [17], and there is CGRP on moderate levels in some brain regions [53]. Additionally, a variety of pathological conditions, including post-herpetic neuralgia and complex region pain syndrome type 1, could drastically up-regulate CGRP in non-sensory neurons [63].

Identification and determination of sensory neuron functional groups were carried out by several approaches. *In vivo* intracellular recording from DRG and TG neurons gives the utmost precision in determining sensory neuronal types [41,42], especially when this method is combined with post-recording IHC [47]. Extracellular recordings of “single fibers” from *ex-vivo* tissue-nerve preparations also produce reliable results [5]. However, these approaches have a problem in visualization of recorded neurons (or fibers) and therefore *ex vivo* electrophysiology approaches combined with ontogenetic are gaining popularity [5]. Accumulated information by *in vivo* intracellular recording makes clear the link between AP/AHP and the corresponding function of sensory neuronal groups disregarding whether neurons innervate paw, vibrissa pad or muscle [22,41,42]. Since AP/AHP parameters generated by patch clamp and intracellular recording are similar [64] (especially at the same recording temperature), whole-cell patch clamp data on isolated DRG (or TG) neurons could be correlated to the function of sensory neuronal groups. Moreover, recordings of responses to a variety of algesic agents, single-cell sequencing information [1,7,12] and detailed characterization of numerous sensory neuronal markers [5,6,7,9,46,47,56,58,65] allow for quick and precise identification of sensory neuronal groups (Table 1). Therefore, this reliable and relatively quick approach can be used in the identification of neuronal groups of molecules/targets that have an appropriate reporter mouse line.

In conclusion, our data show that CGRP^cre-ER/+^ is an effective mouse reporter line for manipulation of C- and A-peptidergic nociceptors. Our data also indicates that targeting nociceptors, but not LTMR neurons can be optimally achieved in TRPV1^cre/+^ reporter mouse lines, while some LTMR neurons could potentially be affected in Nav1.8^cre/+^ mice. Finally, presented here sensory neuron group identification approach could successfully be used for numerous other reporter mouse lines.
